# Engineering electron conduits in bacteria for selective biointerfacing and enhanced energy transfer

**DOI:** 10.1016/j.isci.2026.114805

**Published:** 2026-01-28

**Authors:** Alexander R. Kelly, Lorenzo Travaglini, Dominic J. Glover

**Affiliations:** 1 School of Biotechnology and Biomolecular Sciences, University of New South Wales, Sydney, NSW 2052, Australia

**Keywords:** Bioengineering, microbial biotechnology

## Abstract

Specialized cytochrome protein complexes conduct electrons across cell membranes in electrogenic bacteria, which enables these microbes to be harnessed for applications in electrical generation, biosensing, and microbial electrosynthesis. Here, we engineer the surface-exposed MtrC subunit from the MtrCAB complex of *Shewanella oneidensis* to enable selective cell attachment to functional materials, including electrodes for improved bioelectricity production. Incorporating a SpyTag bioconjugation domain on MtrC enables specific covalent attachment of SpyCatcher-fused proteins to MtrCAB on *S. oneidensis* and *Escherichia coli*. Importantly, the MtrC modification does not disrupt electron export, offering opportunities to interface cells with electronic materials. In the second approach, incorporating a graphite binding sequence on MtrC improves *S. oneidensis* attachment to graphite electrodes, yielding 30% greater current production in a microbial electrolysis cell compared to a variant expressing unmodified MtrC. An engineerable platform on the surface of electrogenic cells creates numerous opportunities for biotic-abiotic interface manipulation.

## Highlights

• *Shewanella oneidensis* uses MtrCAB conduits to move electrons across the outer membrane
• Fusion of SpyTag to the MtrC subunit enabled selective fluorescent bioconjugation
• Graphite binding domain fusion to MtrC improved electrode binding and energy transfer
• Cross-species compatibility of modified MtrC was demonstrated in *Escherichia coli*

## Graphical abstract

**Figure:**
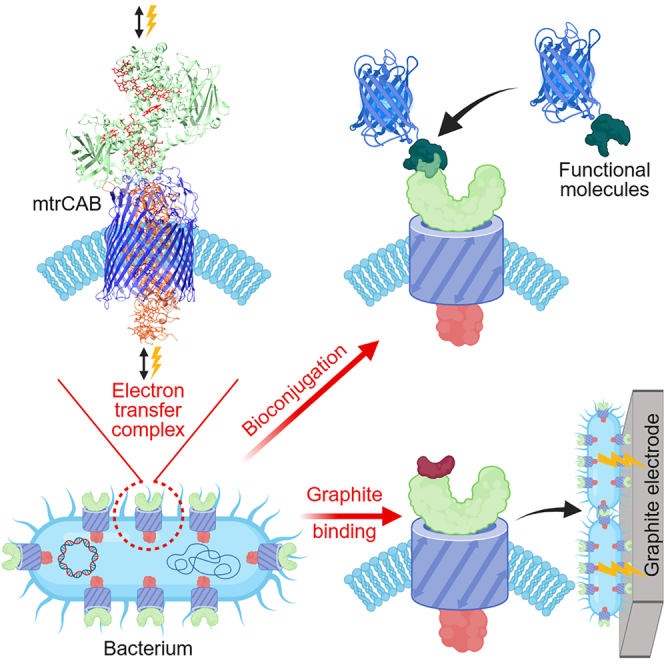

## Introduction

**Figure 1:**
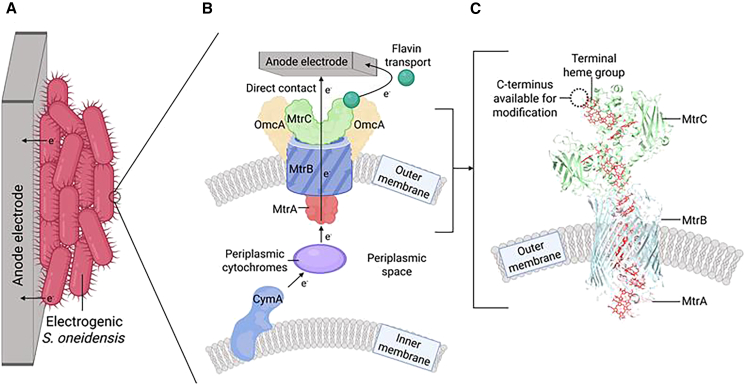
Harnessing electron conduit proteins from *S. oneidensis* for EET (A) Electrogenic bacteria such as *S. oneidensis* can transfer electrons to an anode electrode. (B) Proposed mechanism of EET from within *S. oneidensis* to an extracellular anode. Electrons generated during cellular respiration are transferred from the inner membrane CymA to the outer membrane MtrCAB protein complex through periplasmic cytochromes, with electrons subsequently transferred to an extracellular electrode via direct contact, as well as indirectly through flavin shuttles. (C) Cartoon model view of the X-ray crystal structure of the MtrCAB complex in the outer membrane from *Shewanella baltica.* The decaheme proteins MtrA and MtrC connect through the β-barrel membrane protein MtrB, with the C-terminus of MtrC exposed to the extracellular space. Heme molecules are shown in red. Protein structural model derived from the Protein DataBank (PDB) entry 6R2Q.^5.^

Electrogenic bacteria transfer electrons during anaerobic respiration using specialized cytochrome proteins in their cell membranes to reduce extracellular electron acceptors in their environment, such as metal oxides. The anaerobic respiration of electrogenic bacteria can be harnessed to metabolize waste biomass and transfer electrons to an external electrode, generating electrical energy in a microbial fuel cell (MFC) or hydrogen evolution in a microbial electrolysis cell (MEC).^1^ Beyond energy production, the underlying cellular mechanisms of electron transfer can be used for real-time cellular biosensing,^2^ bioremediation,^3^ or the biosynthesis of valuable chemical and biological products.^4^ These applications rely on the natural capability of electrogenic bacteria to transfer electrons through cell surface electron conduit proteins to or from an electrode, either by physically adhering cells to an electrode or through flavin-mediated extracellular electron shuttling (Figure 1A).^6^ Abiotic modification of electrogenic cells with carbon dots increases extracellular electron transfer (EET) by improving biofilm formation,^7^ and redox-active conjugated oligoelectrolytes enhance microbial electrosynthesis by acting as membrane-integrated redox mediators that mimic transmembrane EET proteins.^8^ Furthermore, coating bacterial cells with a conjugated polyelectrolyte has been shown to boost EET;^9^ however, these polymer synthesis and conjugation steps would be difficult to implement at scale and in a continuous flow MFC/MEC. Engineering cellular EET proteins to improve electrode attachment and electron transfer is an underdeveloped approach that could provide an *in situ* advantage over synthetic techniques. In such an approach, engineered EET proteins localized to the cell surface would function as interfacing or conducting moieties by successive generations of electrogenic cells in a continuous flow MFC/MEC. A variety of surface engineering approaches have been applied to electrogenic bacteria such as *Shewanella oneidensis* to improve surface attachment and enhance EET. In one example, heterologous surface display of a gold-binding peptide on *S. oneidensis* was shown to increase adhesion but displaced outer membrane cytochromes and greatly impaired EET efficiency.^10^ Chemical DNA hybridization has been used to capture *Shewanella* on electrodes, this approach requires complex chemical modification of cells and is challenging to scale for industry.^11^ Addition of glycopolymer-mannose surfaces that exploit pili-lectin interactions can improve binding to gold surfaces, however increased EET was not observed.^12^ In addition, these methods do not directly interface (wire) electron conduit proteins on electrogenic bacteria with electrodes. Directly linking bacterial electron conduit proteins to extracellular materials could enable new approaches to improve EET from microbes to produce renewable energy and enable energy import for biocatalysis and biosensing applications.

The electrogenic bacterium *S. oneidensis* natively couples anaerobic respiration to the reduction of extracellular metals through an EET system that transfers electrons from the cytoplasm to the extracellular space.^13^ Electrons generated during anaerobic metabolism by *S. oneidensis* are passed through the quinone pool to inner membrane electron conduit cytochromes such as CymA (Figure 1B).^14^ Situated on the periplasmic side of the inner membrane, CymA is dynamically responsible for reducing periplasmic cytochromes.^15^ The periplasmic cytochrome proteins, including the small cytochromes CctA and FccA, diffuse through the periplasmic space, carrying electrons to terminal electron acceptors within the periplasm, or to the outer membrane conduit proteins.^16^ The outer membrane electron conduit protein complexes, including MtrCAB and several redundant homologues such as MtrDEF, are responsible for conducting electrons to the extracellular space.^17^ Both MtrA and MtrC are decaheme cytochromes adjoined through the β-barrel membrane-spanning protein MtrB (Figure 1C). Following the delivery of electrons to the MtrA from periplasmic cytochromes, the electrons are transferred through the MtrCAB complex via electron hopping or tunneling between the heme groups.^18^ At the cell surface, electrons are passed to extracellular metals either directly in contact with MtrC or indirectly via secreted flavin electron shuttles and soluble iron.^19^ These flavin shuttles mediate indirect electron transfer, which can be boosted by the addition of artificial redox mediators.^20^ In addition, the outer membrane decaheme cytochrome OmcA may facilitate the diffusion of charge along the *S. oneidensis* outer membrane.^21^ The extracellular electron transport system of *S. oneidensis* can also operate in reverse under aerobic conditions, oxidizing extracellular conductors and possibly using the imported electrons to support metabolism.^22^^,^^23^

The MtrCAB complex is the most extensively characterized component of the EET system, in part due to its role as a transmembrane electron conduit for extracellular electron transfer.^24^ Recombinant expression of the MtrCAB complex in *Escherichia coli* has been demonstrated to convey electrogenic properties to these cells,^25^ especially when co-expressed with the inner membrane conduit CymA,^26^ and a periplasmic shuttle such as STC.^27^ Expression of CymA-MtrCAB in *E. coli* can be linked to intracellular reactions, such as fumarate or nitrate reduction,^28^ expanding the range of tools available for microbial electrosynthesis.^29^ The addition of further recombinant pathways created an *E. coli-based* biosensor that responded to environmental contamination with an electrical output.^2^ An X-ray crystallography-resolved structure of the MtrCAB complex from the related species *S*. *baltica* revealed that the C-terminus of MtrC is exposed to the extracellular space, offering a promising site for genetic modification (Figure 1C).^5^

Here, we demonstrate that the MtrCAB electron conduit complex of *S. oneidensis* is directly amenable to protein engineering to enable selective and modular attachment of cells to functional materials without disrupting EET. In this approach, extracellular-facing cytochrome MtrC was modified in the native host *S. oneidensis* and recombinantly in *E. coli* to enable the attachment of functional domains or materials. The addition of a SpyTag domain to MtrC enabled the bioconjugation of fluorescent proteins without impeding the EET capability of the host. Furthermore, the addition of the SpyTag cell-surface bioconjugation site to the MtrCAB electron-export conduit facilitated relative quantification of MtrCAB expression and could potentially provide a platform for redox biochemistry powered by cellular metabolism. Fusion of the graphite binding peptide GrBP5^30^ to MtrC resulted in improved attachment of *S. oneidensis* to graphite electrodes and a significant increase in current generation in an MEC. The design and engineering of conduit-level interfaces rather than surface decoration overcomes previous reports of disrupted EET, and can serve as a platform for the future attachment of conductive proteins or redox enzymes to the cell surface for the transport or utilization of exported electrons.^31^

## Results and discussion

### Engineering of MtrC for the attachment of functional domains

Modification of the extracellular-facing side of the MtrCAB complex could enable improved binding to different electrode materials in MFCs/MECs for improved electron transfer or for the attachment of functional molecules, such as nanoparticles for electrochemical reactions^32^ or biocatalysis.^33^ We hypothesized that the C-terminus of the MtrC should be amenable to protein engineering to attach functional domains without negatively impacting cellular electron transfer capability. Furthermore, the incorporation of a bioconjugation tag to the C-terminus of MtrC should enable specific attachment of functional molecules and serve as a modular platform to modify the cell surface MtrCAB complex. The SpyTag-SpyCatcher system was chosen to enable rapid and specific bioconjugation of proteins to the MtrC. In this bioconjugation system, the SpyTag peptide spontaneously forms an intermolecular isopeptide bond with the SpyCatcher protein.^34^ A fusion protein was designed with the SpyTag attached to the C-terminus of MtrC through a flexible linker region to prevent the SpyTag from interfering with MtrC folding, heme incorporation, and association with MtrA and MtrB (Table S1). Furthermore, the flexible linker should improve the extracellular exposure of the SpyTag domain and prevent steric hindrance for the attachment of a SpyCatcher-functionalized protein. Protein structural modeling using the AlphaFold 3 server^35^ suggested that a GSGESGSG (single letter amino acid code) linker between the C-terminal of MtrC and the SpyTag sequence would provide sufficient space for the attachment of SpyCatcher fused to a fluorescent protein (Figure S1A and Table S1).

The MtrC-SpyTag was engineered to be recombinantly expressed in an *S. oneidensis* strain that has had its outer membrane conduits *mtrC*, *mtrF,* and *omcA* knocked out (*S. oneidensis* Δ*mtrC*Δ*mtrF*Δ*omcA*) and in the *E. coli* BL21(DE3) strain (Table S2). The two different bacterial species provide different advantages to express MtrC-SpyTag for bioconjugation, with *S. oneidensis* offering superior exoelectrogenic characteristics over *E. coli*,^26^ while *E. coli* has optimized tools for genetic engineering and protein expression, as evidenced by its use in building biosensors for the bioelectronic sensing of environmental contaminants.^2^ A variety of ribosome binding site (RBS) strengths have been used previously for the expression of MtrC in *S. oneidensis,* with a stronger RBS encouraging higher MtrC expression at the cost of exoelectrogenic activity.^36^ Plasmids were constructed to express MtrC-SpyTag in *S. oneidensis* with either a weak RBS (pCD24r1) or a strong RBS (pCD24r6) regulated by an IPTG-inducible promoter. As *E. coli* lacks an endogenous MtrCAB complex, we utilized a previously developed system for the recombinant expression and maturation of CymA, MtrC, MtrA and MtrB assisted by the co-expression of the pEC86 periplasmic heme maturation plasmid (Table S3) in *E. coli* BL21(DE3) (Table S2).^25^^,^^26^ A plasmid previously constructed to express the CymA and MtrCAB was modified to include SpyTag in fusion with the MtrC, with expression of these genes regulated by an IPTG-inducible T7 promoter and strong RBS (Table S3).

**Figure 2:**
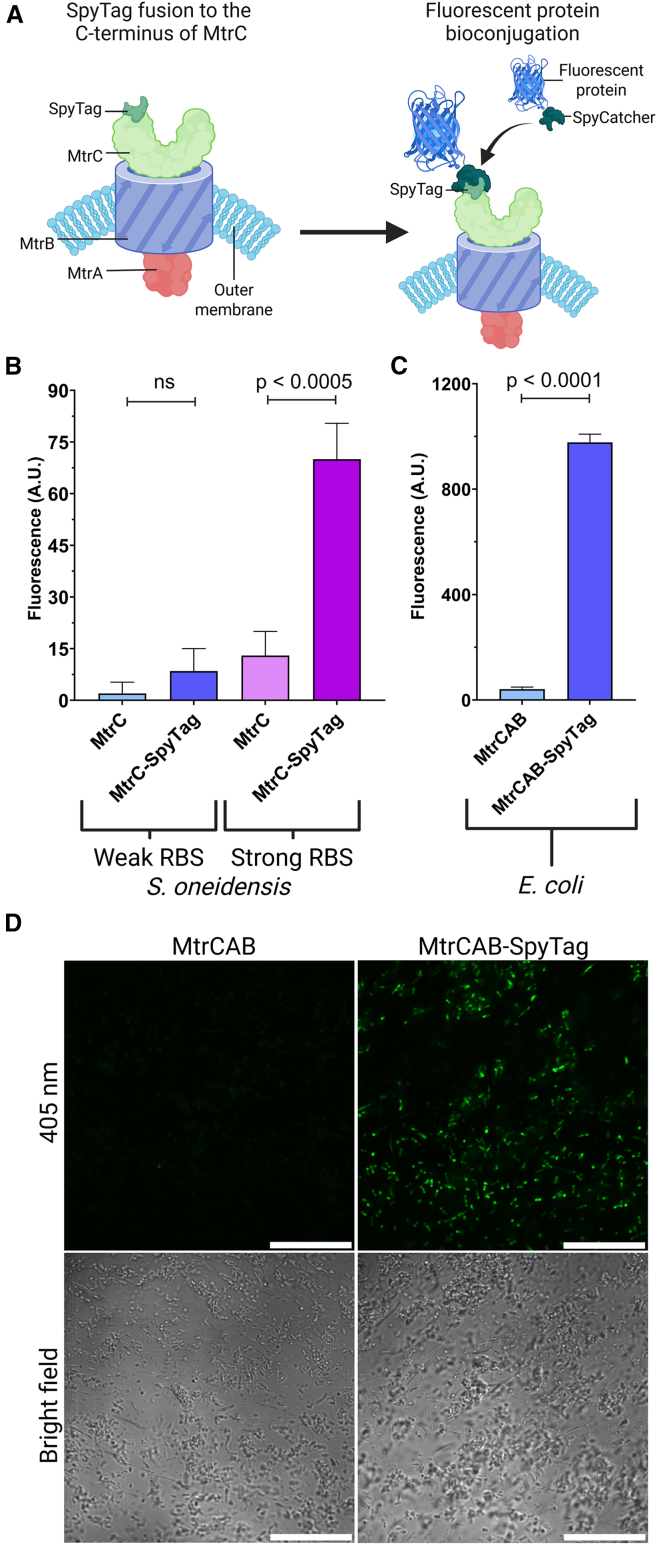
Functional modification of MtrC with a SpyTag bioconjugation domain in *S. oneidensis* and *E. coli* (A) Fusion of a SpyTag domain to the C-terminus of MtrC enables the bioconjugation of a SpyCatcher-fused fluorescent protein, facilitating selective surface labeling of modified cells. (B) The fluorescence of *S. oneidensis* cells was quantified by spectrofluorometry (arbitrary units) and shown as the mean of 3 separate experiments performed in triplicate with subtracted background fluorescence of unreacted control cells. Error bars represent standard deviation between experiments (*n* = 3, significance calculated using a parametric, unpaired, one-tailed *t* test). Expression of MtrC or MtrC-SpyTag in *S. oneidensis* cells was regulated using either a weak or strong RBS. (C) The fluorescence of *E. coli* cells expressing CymA-MtrCAB or CymA-MtrCAB-SpyTag shown as the mean of 3 separate experiments performed in triplicate, with subtracted background fluorescence of unreacted control cells. Error bars represent standard deviation between experiments (*n* = 3, significance calculated using a parametric, unpaired, one-tailed *t* test). (D) Fluorescence microscopy revealed observable mCerulean3 fluorescence for *E. coli* cells expressing CymA-MtrCAB-SpyTag compared with cells expressing CymA-MtrCAB following incubation with mCerulean3-SpyCatcher. Scale bars, 100 μm.

The recombinant expression and exposure of the MtrC-SpyTag on the surface of *S. oneidensis* and *E. coli* cells was examined by measuring the bioconjugation of a fluorescent protein, mCerulean3, that was fused to SpyCatcher (Figure 2A). The mCerulean3-SpyCatcher fusion protein contains an N-terminal hexahistidine tag for protein purification by immobilized metal affinity chromatography, followed by mCerulean3 and the SpyCatcher domain, with GSG linkers included between the constitutive elements to ensure flexibility and mitigate folding disruption (Table S1). Cultures of *S. oneidensis* cells induced to express MtrC or MtrC-SpyTag using either the weak RBS (pCD24r1) or a strong RBS (pCD24r6) were incubated with mCerulean3-SpyCatcher to promote bioconjugation. Subsequently, the cells were washed extensively to remove unbound fluorescent reporter protein, and cell fluorescence was quantified by spectrofluorometry. A statistically significant 5-fold increase in mCerulean3 fluorescence was observed for *S. oneidensis* cells engineered to express the MtrC-SpyTag under the strong RBS compared to unmodified MtrC, while no significant increase in fluorescence was observed for the weak RBS (Figure 2B). These results suggest the SpyTag domain is available for bioconjugation when MtrC-SpyTag is expressed under the control of a strong RBS, while the weak RBS system did not produce sufficient surface-exposed MtrC-SpyTag for mCerulean3-SpyCatcher fluorometric detection. Expression of MtrC under the control of the strong RBS has been shown previously to have diminished electrogenic properties compared to the weak RBS.^36^ We confirmed that the strong RBS produces less current than the weak RBS in a microbial electrolysis cell (MEC) (Figure S2). This reduced electrogenic activity may be due to an introduced bottleneck, such as an overloading of the periplasmic heme incorporation machinery. Next, we examined if recombinant expression of the full MtrCAB operon in *S. oneidensis* JG1194 (knockout of *mtrC*, *omcA*, *mtrF*, *mtrA*, *mtrD*, *dmsE*, *so4360*, *cctA,* and *recA*; Table S2) using the weak RBS would produce more surface-exposed MtrC-SpyTag for mCerulean3-SpyCatcher bioconjugation. However, the expression of the complete MtrCAB-SpyTag did not significantly improve fluorophore binding (Figure S3A). An alternative explanation was that the lack of fluorescence following mCerulean3-SpyCatcher bioconjugation to *S. oneidensis* was due to fluorescent quenching by the many heme groups in MtrC proteins. The emission properties of mCerulean3 are similar to EGFP, which has been demonstrated to be susceptible to heme quenching. Other fluorescent proteins, such as mKate2, that possess a red emission spectrum that does not overlap with the absorption spectra of heme, avoid being fluorescently quenched by heme.^37^ The effect of heme-mCerulean3 fluorescent quenching was examined by replacing the mCerulean3 with mKate2 in fusion with SpyCatcher domain. Reacting mKate2-SpyCatcher with *S. oneidensis* cells expressing MtrC-SpyTag via the strong pCD24r6 promoter resulted in a 2.8-fold-change in fluorescence (Figure S3B). The mKate2 bioconjugation thus produced less fluorescence and a smaller fold change than the mCerulean3 bioconjugation, indicating that heme quenching was not measurably affecting the measured cell fluorescence. The expression and surface exposure of SpyTag in the following engineered *E. coli* strains proved to be a more robust platform for the bioconjugation of the SpyCatcher-tagged fluorescent protein. Incubation of mCerulean3-SpyCatcher with *E. coli* cells that express the CymA-MtrCAB-SpyTag produced a 24-fold increase in fluorescence compared to cells expressing CymA-MtrCAB (Figure 2C). Furthermore, this efficient labeling of MtrC-SpyTag on *E. coli* cells was able to be visualized by fluorescence microscopy, with *E. coli* cells engineered to express CymA-MtrCAB-SpyTag shown to be fluorescent in comparison to the CymA-MtrCAB *E. coli* strain (Figure 2D).

The amount of fluorescent cell labeling of *S. oneidensis* and *E. coli* is presumably related to the level of expressed and surface-localized MtrC-SpyTag. The strong RBS in *S. oneidensis* and the high-activity T7 expression system in *E. coli* most likely produce many copies of the MtrC-SpyTag protein, which are displayed on the cell surface. However, as shown in the MEC chronoamperometry experiments (Figure S2), and in previous research,^36^ high expression of MtrC produces *S. oneidensis* with reduced EET ability. It has been hypothesized that the poor EET after IPTG induction of the strong RBS in the *S. oneidensis* system may be due to bottlenecks in the post-translational processing steps required to produce functional MtrC protein.^36^ Overloading of the heme incorporation machinery could produce non-functional MtrC, which nonetheless is incorporated into MtrCAB complexes and presents SpyTag domains for binding. Conversely, the expression of MtrC-SpyTag via the weak RBS likely results in functional MtrCAB-SpyTag complexes on the cell surface, but at copy numbers too low for detection with the mCerulean3-SpyCatcher probe. Conjugating SpyCatcher to brighter fluorophores, such as quantum dots,^21^ may enhance the detection of the MtrC-SpyTag on the cell surface. Regardless, the expression of MtrC-SpyTag via the weak RBS and its incorporation into MtrCAB on the cell surface should be verifiable by assessing whether these engineered *S. oneidensis* exhibit electroactivity.

### Validation of extracellular electron transfer capacity by the engineered MtrCAB complexes

**Figure 3:**
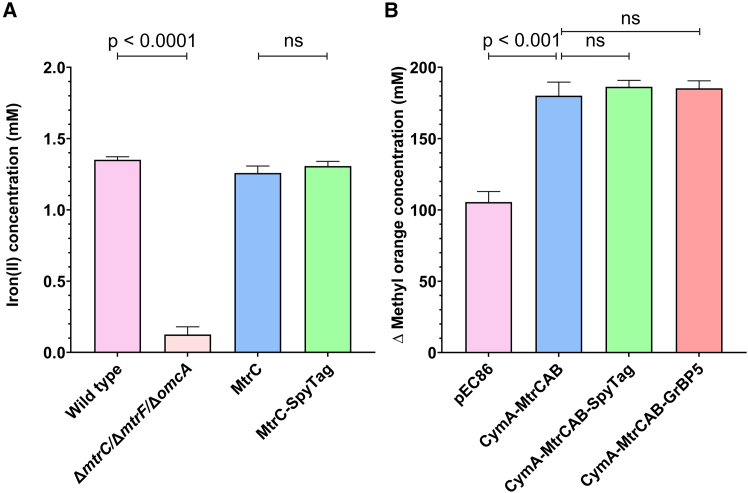
Extracellular substrate reduction by *S. oneidensis* and *E. coli* cells that express engineered MtrC proteins (A) Reduction of iron(III) to iron(II) by wild-type *S. oneidensis*, compared to a Δ*mtrC*/Δ*mtrF*/Δ*omcA* knockout strain. The Δ*mtrC*/Δ*mtrF*/Δ*omcA* knockout strain was subsequently engineered to express MtrC or MtrC-SpyTag. The formation of iron(II) after 16 h of anaerobic culture was quantified using a ferrozine assay and shown as a mean of three experiments with three biological repeats. Error bars represent standard deviation between experiments (*n* = 3, significance calculated using a parametric, unpaired, two-tailed *t* test). (B) Reduction of methyl orange with electrons from *E. coli* BL21(DE3) cells containing only the pEC86 plasmid, or cells co-expressing either the CymA-MtrCAB complex, CymA-MtrCAB with SpyTag, or CymA-MtrCAB with GrBP5. The change in methyl orange absorbance at 465 nm upon the reduction of the azo group was measured after 24 h of anaerobic culture, and is shown as a mean of three experiments with three biological repeats (*n* = 3, significance calculated using a parametric, unpaired, two-tailed *t* test). Error bars represent the standard deviation between experiments.

After demonstrating that the MtrC can be modified in both *E. coli* and *S. oneidensis* to display a surface bioconjugation domain, we examined whether these cells remained electroactive and capable of electron export to extracellular metal acceptors. The ferrozine-based colorimetric assay is a common approach to measure the bacterial reduction of iron(III) to iron(II).^36^ The ferrozine assay was validated by comparing the difference between the wild type strain of *S. oneidensis* and the Δ*mtrC*/Δ*mtrF*/Δ*omcA* knock out strain of *S. oneidensis* that served as a negative control (Figure 3A). Subsequently, the iron-reducing capacity of *S. oneidensis* cells expressing recombinant MtrC or MtrC-SpyTag under the control of the weak pCD24r1 RBS was measured. The ferrozine assay revealed that fusing a SpyTag domain to MtrC did not significantly impede the ability of *S. oneidensis* cells to reduce extracellular iron(III) (Figure 3A). Importantly, this assay also confirmed that the MtrC-SpyTag was expressed via the weak RBS and incorporated into the MtrCAB complex on the cell surface to enable EET.

The EET capacity of recombinantly expressed CymA-MtrCAB in *E. coli* was measured using a methyl orange reduction assay, in which the reduction of the azo group of methyl orange results in a clear colorimetric change at 465 nm.^27^ The inefficient exoelectrogenic properties of the recombinant *E. coli* are likely due to an incomplete transfer of the EET machinery. Recent research published during the development of our system demonstrated improved EET in *E. coli* via the introduction of heme-containing periplasmic cytochromes to help bridge the gap between CymA and MtrCAB.^27^ Using a methyl orange assay to measure electron export, we demonstrated that *E. coli* cells expressing CymA-MtrCAB with the aid of the pEC86 heme-maturation proteins were significantly better at reducing methyl orange compared to *E. coli* cells expressing heme-maturation proteins only (Figure 3B). Furthermore, there was no significant difference between the CymA-MtrCAB, CymA-MtrCAB-SpyTag, and CymA-MtrCAB-GrBP5 strains, indicating a similar capacity for EET (Figure 3B).

### Enhanced electrode binding and extracellular electron transfer capacity by engineering MtrC with a graphite binding domain

**Figure 4:**
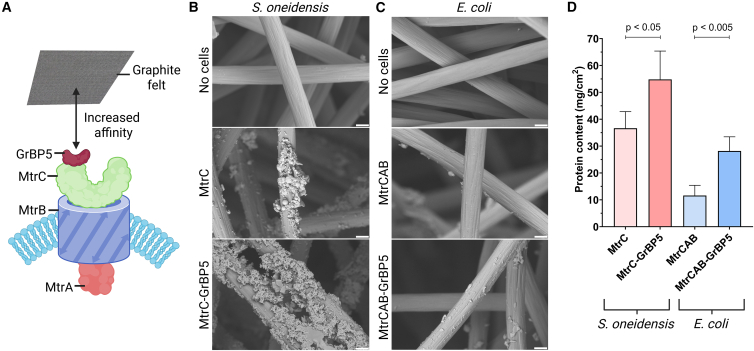
Improved graphite binding by *S. oneidensis* and *E. coli* cells expressing MtrC-GrBP5 (A) Illustration shows the C-terminal modification of MtrC with a graphite binding domain to improve the affinity of *S. oneidensis* cells to a graphite electrode. (B) SEM images of graphite felt fibers with no cells, as well as with bound *S. oneidensis* Δ*mtrC*/Δ*mtrF*/Δ*omcA* cells expressing either MtrC or MtrC-GrBP5 (scale bars, 5 μm). (C) SEM images of graphite felt fibers with no cells, as well as with bound *E. coli* BL21(DE3) expressing either CymA-MtrCAB or CymA-MtrCAB-GrBP5 (scale bars, 5 μm). (D) Lysed protein content from a graphite felt electrode aerobically incubated with *S. oneidensis* or *E. coli* cells expressing MtrC with or without the GrBP5 binding domain. The data represent the mean protein per cm^2^ of geometric surface area of graphite felt electrode (mg/cm^2^) in cell lysate from three biological replicates from three separate experiments of *S. oneidensis* Δ*mtrC*/Δ*mtrF*/Δ*omcA* cells expressing recombinant MtrC or MtrC-GrBP5, or of *E. coli* BL21(DE3) cells expressing CymA-MtrCAB-GrBP5 compared to CymA-MtrCAB. Error bars represent standard deviation between experiments (*n* = 3, significance calculated using a parametric, unpaired, one-tailed *t* test).

Following the proof of concept to engineer MtrC with SpyTag, we sought to modify MtrC to improve the binding affinity of bacterial cells to a graphite electrode and serve as an engineered biotic-abiotic interface to improve current production in an MEC.^38^ The culturing of electroactive organisms enables renewable energy or hydrogen to be produced while simultaneously bioremediating wastewater, however challenges in industrial scalability have prevented widespread adoption.^39^ Biofilms formed by *S. oneidensis* grown on graphite felt at 0 V (205 mV vs. SHE) have been observed to be thin and uneven, suggesting improved cellular electrode attachment could increase current production.^40^ We hypothesized that engineering the extracellular side of MtrC with the graphite binding protein GrBP5 would improve cell adhesion to graphite electrodes and promote biofilm formation. Furthermore, this MtrC modification should improve electrode colonization, and consequently, accelerate and increase current generation in MECs (Figure 4A). The graphite binding protein GrBP5 was previously identified by phage display and possesses micromolar affinity for graphite surfaces.^41^ The MtrC-SpyTag fusion protein was used as a model for GrBP5 addition, with the SpyTag domain replaced with the GrBP5 sequence in both the *S. oneidensis* and *E. coli* expression plasmids, while maintaining the GSGESGSG linker between the C-terminal of MtrC and the GrBP5 domain (Figure S1B and Table S1).

The graphite binding capacity of the MtrC-GrBP5 modified *S. oneidensis* and *E. coli* cell strains was examined by scanning electron microscopy (SEM). Aerobically cultured *S. oneidensis* cells expressing MtrC-GrBP5 under control of the weak RBS possessed greater observable binding and biofilm accumulation on the fibers of graphite felt compared to unmodified MtrC expressing cells (Figure 4B). Likewise, after aerobic incubation with graphite felt, *E. coli* cells expressing CymA-MtrCAB-GrBP5 were observed by SEM at a greater density on the graphite fibers compared to CymA-MtrCAB expressing *E. coli* (Figure 4C). Improved graphite binding by MtrC-GrBP5 modified cells was quantified by rinsing the graphite felt to remove weakly bound cells and culture components, and lysing the cells on the graphite to measure total protein content. A significant increase in lysed protein content was observed for *S. oneidensis* cells expressing MtrC-GrBP5 under the control of a weak RBS compared to cells expressing MtrC (Figure 4D). Similarly, *E. coli* expressing CymA-MtrCAB-GrBP5 had over twice the amount of protein content in cell lysate compared to the CymA-MtrCAB strain (Figure 4D).

The graphite binding assay suggests that the expression of MtrC-GrBP5 in both *E. coli* and *S. oneidensis* resulted in increased affinity for the graphite felt and greater cell attachment. When comparing the two species, *S. oneidensis* expressing unmodified MtrC or MtrC-GrBP5 was shown by SEM imaging and the total protein assay to attach to the graphite at a higher density than either of the *E. coli* strains. This is presumably due to the *S. oneidensis* cells having a stronger natural affinity for graphite felt compared to *E. coli*, further investigation would be necessary to validate this theory. Furthermore, the weaker natural interaction for *E. coli* on graphite could have produced the greater fold-change in electrode binding when expressing the MtrC-GrBP5. In addition, the larger fold-change observed for the *E. coli* strain may have resulted from greater expression and surface display of the MtrC-GrBP5 compared to the modified *S. oneidensis*, in a similar manner as the greater MtrC-SpyTag surface density observed in the fluorescent bioconjugation experiments (Figure 2).

**Figure 5:**
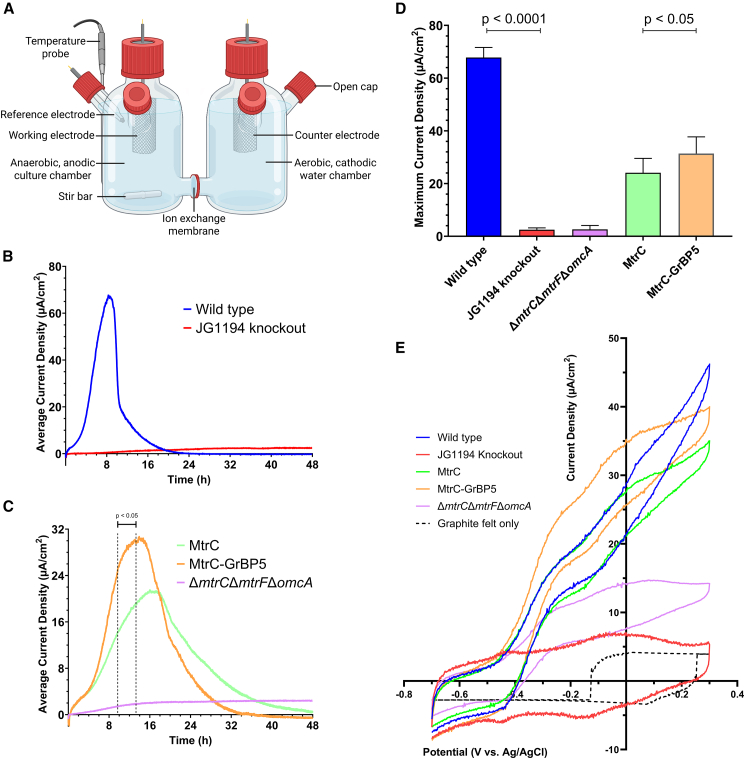
Fusion of a GrBP5 domain to MtrC results in an accelerated and higher peak of current generation during MEC chronoamperometry of an *S. oneidensis* cell culture (A) MEC setup for chronoamperometry experiments with graphite felt serving as working and counter electrodes, alongside an Ag/AgCl reference electrode. (B) Chronoamperometry of wild-type *S. oneidensis* and *S. oneidensis* JG1194 negative control shown as the mean of three repeats. (C) Chronoamperometry of *S. oneidensis* Δ*mtrC*/Δ*mtrF*/Δ*omcA* cells (mean of three repeats) or this strain modified to recombinantly express MtrC or MtrC-GrBP5 (mean of five repeats). Fusion of a GrBP5 binding domain to the C-terminus of MtrC results in an accelerated increase in current density and higher maximum current density (μA/cm^2^) by *S. oneidensis* cells. Region with significant difference calculated based upon standard deviation (*n* = 5, significance calculated using a parametric, unpaired, one-tailed *t* test). (D) Histogram comparing the mean maximum current density (μA/cm^2^) of cells grown in MEC from the chronoamperometry shown in B and C. Error bars indicate standard deviation (*n* = 3 [Wild type, JG1194, Δ*mtrC*/Δ*mtrF*/Δ*omcA*] or *n* = 5 [MtrC, MtrC-GrBP5], significance calculated using a parametric, unpaired, one-tailed *t* test). (E) Cyclic voltammetry of each cell strain after 24 h of biofilm establishment and 6 h of growth in fresh media compared with bare carbon felt.

After demonstrating improved electrode attachment by the incorporation of the GrBP5 domain on MtrC, we examined whether these functionalized cells would enhance the maximum current density produced in an MEC. In an MEC, electrodes are separated into two cells separated by a cation exchange membrane. Exoelectrogenic bacteria metabolize an organic substrate at the anode with an applied potential to catalyze hydrogen evolution at the cathode.^42^ An MFC uses the same setup but with an external resistor instead of an applied potential, generating electricity from substrate oxidation coupled to oxygen reduction at the cathode.^43^ Cultured microorganisms in MFCs/MECs can simultaneously remove pollutants from landfills^44^^,^^45^ or wastewater^46^^,^^47^ while generating electricity or hydrogen. A two-chamber MEC was constructed that consisted of a 4.5 cm × 3.5 cm x 0.28 cm graphite felt working electrode (AvCarb G280A) and Ag/AgCl reference electrode (Xi’an Yima Opto-electrical Technology) in one chamber, and a matching graphite felt counter electrode in the second chamber, with the chambers separated by a cation exchange membrane (Aquivion E98-15S) (Figures 5A and S4). MEC functionality was confirmed using chronoamperometry with an applied potential of 0 V vs. Ag/AgCl reference (205 mV vs. SHE) to compare a positive control of wild-type *S. oneidensis* to a negative control of *S. oneidensis* JG1194 (Figure 5B). The wild-type *S. oneidensis* produced a mean maximum current density of 67.8 μA/cm^2^ that was produced at a mean time of 8.5 h, whereas the knockout strain produced minimal current over the 48 h of chronoamperometry. The observed current density of the WT *S. oneidensis* in our system is consistent with previously reported MECs operated under similar conditions.^48^^,^^49^ Chronoamperometry was used to compare the current generation of *S. oneidensis* Δ*mtrC*/Δ*mtrF*/Δ*omcA* cells recombinantly expressing MtrC or MtrC-GrBP5. A negative control of *S. oneidensis* Δ*mtrC*/Δ*mtrF*/Δ*omcA* cells containing no plasmid displayed a similar lack of performance as the JG1194 negative control (Figure 5C). Cells expressing the fusion of GrBP5 to MtrC were shown to generate a higher maximum current density of 31.4 μA/cm^2^ that peaked at an earlier time compared to *S. oneidensis* cells expressing unmodified MtrC (Figure 5C), which produced a mean peak of 24.1 μA/cm^2^. The mean current density of the two recombinant systems was similar until the 5-h mark, after which the current generation by MtrC-GrBP5 was shown to be significantly improved from 9.7–13.2 h. The MtrC-GrBP5 cell strain achieved a 30% higher mean maximum current density than unmodified MtrC (Figure 5D), this was significantly lower than the wild type *S. oneidensis*. The modified MtrC-GrBP5 strain has additional EET proteins knocked out, including *mtrF* and *omcA*, which likely accounts for the lower EET efficiency. In the future, the wild-type *S. oneidensis* could be genomically modified to observe whether MtrC-GrBP5 fusion improves the current generation for cells containing the full complement of outer membrane conduit machinery. Cyclic voltammetry (CV) revealed that MtrC-GrBP5-expressing cells exhibited similar redox behavior to WT and unmodified MtrC strains, confirming functional electron transfer through the engineered MtrC (Figure 5E). In contrast, the knockout strain and bare carbon felt controls showed minimal redox response, further supporting the importance of MtrC in mediating electron transfer under these conditions. SEM imaging of the graphite felt electrode of MECs culturing *S. oneidensis* Δ*mtrC*/Δ*mtrF*/Δ*omcA* cells after 11.5 h of operation revealed an uneven distribution of cells, which prevented unbiased quantification (Figure S5).

MFC and MEC technologies have shown promise in the bioremediation of wastewater, with electricity or hydrogen generation as a useful byproduct,^50^ as well as applications in microbial electrosynthesis^51^ and biosensing.^2^ By demonstrating that MtrC can be functionally modified to boost MEC performance, this research opens up new avenues of investigation in the fields of bioremediation, microbial electrosynthesis, biosensing, and energy harvesting. Although a 30% increase in current generation is a modest improvement over the unmodified MtrC, this result suggests that the biological engineering of the biotic-abiotic interface in an MEC can improve efficiency. Furthermore, engineering the MtrC with specific attachment domains lays the foundation to directly wire materials, such as synthetic conductive polymers,^8^^,^^9^ with microbial electron conduits.

### Concluding remarks

Taken in conjunction, the bioconjugation, electroactivity, and graphite binding results reveal that functional modifications can be made to the C-terminus of MtrC without significantly impeding the exoelectrogenic properties of the MtrCAB complex. The engineering of the biotic-abiotic interface to encourage favorable electrode binding dynamics offers a promising direction to improve MFC/MEC efficiency for renewable energy generation and bioremediation, which could be implemented in emerging continuous MFC/MEC configurations with artificial redox mediators.^20^ Targeting a C-terminal MtrC modification to artificial redox mediators could further boost the efficiency of these continuous flow reactors. SpyTag-SpyCatcher bioconjugation of outer-membrane proteins may also facilitate patterned biofilm formation for improved current output in a heterogeneous coculture with sulfate-reducing bacteria.^52^

Previous research has demonstrated that electron export from electroactive bacteria can be enhanced by linking cells to electrodes through conductive polymers or nanomaterials.^53^^,^^54^ These strategies may be further improved by engineering MtrCAB to enable specific, spatially controlled attachment to electrode materials. For example, fusion to MtrC SpyTag could allow targeted bioconjugation to complementary functional moieties containing SpyCatcher, such as nanoparticles, quantum dots, or enzymes, facilitating modular interfacing with the cell surface.^21^ In such configurations, MtrCAB could act as an electron donor to the conjugated moiety, supporting applications in biosensing or catalysis.

Beyond individual protein conjugation, incorporating SpyTag-modified MtrC into conductive biofilms containing SpyCatcher may promote tighter adhesion of cells to electrodes in MFCs and MECs, potentially enhancing electron transfer efficiency. Moreover, introducing alternative bioconjugation domains into the MtrDEF complex could enable selective wiring of extracellular electrodes to inner membrane redox proteins, creating opportunities for multi-channel redox communication.^55^ As high-density electrode arrays continue to miniaturize toward the nanometer scale, bioelectronic interfaces will increasingly operate at the resolution of individual proteins.^56^ A protein-level, engineerable interface that is enabled by synthetic surface display systems such as SpyTag-SpyCatcher could allow for the precise attachment of cells to individual electrodes, opening the door to multiplexed electrical interrogation of intracellular processes.

### Limitations of the study

Expression of MtrC-GrBP5 in *S. oneidensis* under the control of the strong RBS was not examined, as MEC chronoamperometry experiments for the recombinant expression of MtrC (Figure S2) revealed that the greater expression of the MtrC-GrBP5 may result in cells with poor EET capability. This result has implications for the recombinant expression of the MtrCAB complex in *E. coli*, which is controlled by the T7 expression system with a high-strength RBS.^25^^,^^26^ Comparing the fold change in fluorophore binding by the *E. coli* and *S. oneidensis* bioconjugation systems in this research, there appears to be far more MtrCAB complexes being expressed in the recombinant *E. coli* than in the optimized *S. oneidensis* system. It is possible that a less-is-more approach to the engineering of recombinant electroactive bacteria may yield improved exoelectrogenic outcomes. An important caveat to this research is that the recombinant electroactive strains did not outperform the WT organism in the MEC. Genome-level modification of the wild-type *S. oneidensis* could be performed in the future to fuse GrBP5 to MtrC for expression using the native regulatory system. Importantly, this would enable the assessment of the effects that the OmcA and MtrF surface EET proteins would have on cellular electrode binding by the MtrC-GrBP5. Both the *omcA* and *mtrF* genes were knocked out in the strains used to examine enhanced electrode and energy production by the recombinantly expressed MtrC-GrBP5. The OmcA is especially relevant as this protein attaches in a 2:1 complex with MtrC,^57^ which may affect access to the C-terminus of MtrC. This study does not feature MEC data for the engineered *E. coli* strains, as we were unable to generate more current with the CymA-MtrCAB expressing strains than an unmodified control.

## Resource availability

### Lead contact

Further information and requests for resources should be directed to and will be fulfilled by the lead contact, Dominic J. Glover (d.glover@unsw.edu.au).

### Materials availability

• All chemicals were purchased from commercial resources and used as received.
• Plasmid DNA, plasmid DNA sequences, and cell strains created in this study are available from the lead contact upon request.

### Data and code availability

• Data reported in this study will be shared by the lead contact upon reasonable request.
• This study did not generate new code.
• Any additional information required to reanalyze the data reported in this study is available from the lead contact upon request.

## Acknowledgments

Research was sponsored by the Office of Naval Research Global and was accomplished under Award Number: N62909-21-1-2019. The authors acknowledge the assistance and facilities of the Mark Wainwright Analytical Centre, including the Electron Microscopy Unit for SEM. We thank the UNSW Recombinant Products Facility and the UNSW Structural Biology facility for access to protein purification equipment. We appreciate the Katharina Gaus Light Microscopy Facility for its assistance in fluorescent microscopy imaging. We thank Benjamin Keitz (10.13039/100008562University of Texas at Austin) for the *S. oneidensis* expression plasmids, and Jeffrey Gralnick (10.13039/100007249University of Minnesota) and Benjamin Keitz for the *S. oneidensis* knockout strains. We thank Caroline Ajo-Franklin (10.13039/100007863Rice University) for the *E. coli* MtrCAB expression plasmids. We appreciate helpful advice from Sarah Glaven (10.13039/100025807Naval Research Laboratory) on MEC construction and operation. A.R.K. is supported by an Australian Government Research Training (RTP) Scholarship. Diagrams were produced using BioRender (https://www.biorender.com/).

## Author contributions

A.R.K designed experimental procedures, designed, built, and characterized plasmids and proteins, performed fluorescence, ferrozine, and graphite binding assays, built and operated the MECs, prepared SEM samples, and wrote the article with input from all authors. L.T. designed, and troubleshooted MEC operation, and performed SEM of the graphite felt. D.J.G. designed and supervised the experimental procedures and edited the article.

## Declaration of interests

The authors declare no competing interests.

## STAR★Methods

### Key resources table

**Table:** 

REAGENT or RESOURCE	SOURCE	IDENTIFIER
**Bacterial and virus strains**		

*E. coli* NEB Turbo	NEB	Cat#C2987
*E. coli* BL21 (DE3)	NEB	Cat#C2527
*S. oneidensis* MR-1 WT	Myers and Nealson 1988, University of Wisconsin, Milwaukee^63^	–
*S. oneidensis* Δ*mtrC*/Δ*mtrF*/Δ*omcA* JG596	Coursolle & Gralnick 2010, University of Minnesota, Twin Cities^62^	JG596
*S. oneidensis* JG1194	Coursolle & Gralnick 2012, University of Minnesota, Twin Cities^60^	JG1194

**Chemicals, peptides, and recombinant proteins**		

mCerulean3-SpyCatcher	This study	–
mKate2-SpyCatcher	This study	–
Potassium chloride	Sigma Aldrich	Cat#P3911
Tryptone (Bacteriological)	Oxoid	Cat#LP0042B
Yeast extract	Thermo scientific	Cat#468550010
Sodium chloride	Sigma Aldrich	Cat#S9888
Agar	Sigma Aldrich	Cat#A1296
Potassium phosphate monobasic	Sigma Aldrich	Cat#P5655
Potassium phosphate dibasic	Sigma Aldrich	Cat#P3786
Magnesium sulfate heptahydrate	Sigma Aldrich	Cat#230391
Ammonium sulfate	Sigma Aldrich	Cat#A4915
HyCase SF casamino acids	Sigma Aldrich	Cat#C9386
HEPES	Sigma Aldrich	Cat#H3375
Trace mineral supplement	ATCC	Cat#MD-TMS
Sodium L-lactate	Sigma Aldrich	Cat#L7022
Sodium fumarate	Sigma Aldrich	Cat#F1506
Isopropyl β-*d*-1-thiogalactopyranoside (IPTG)	Sigma Aldrich	Cat#I5502
Ampicillin, sodium salt	Sigma Aldrich	Cat#A9518
Kanamycin sulfate	Sigma Aldrich	Cat#K1377
Chloramphenicol	Sigma Aldrich	Cat#C0378
Imidazole	Sigma Aldrich	Cat#I202
HisPur Ni-NTA resin	Thermo Fisher	Cat#88223
Phosphate buffered saline	This study	–
Glycerol	Sigma Aldrich	Cat#G9012
Iron(III) citrate	Sigma Aldrich	Cat#F3388
Iron(II) sulfate heptahydrate	Sigma Aldrich	Cat#F7002
3-(2-Pyridyl)-5,6-diphenyl-1,2,4-triazine-p,p’-disulfonic acid monosodium salt hydrate (ferrozine)	Sigma Aldrich	Cat#160601
Methyl orange	Sigma Aldrich	Cat#1013220025

**Critical commercial assays**		

Bradford assay	Bio-Rad	Cat#5000006
Miniprep plasmid purification	QIAGEN	Cat#12125
Gibson assembly master mix	NEB	Cat#E2611S
NEBridge Golden Gate assembly kit (BsaI-HF v2)	NEB	Cat#E1601S
Q5 High-Fidelity 2X master mix	NEB	Cat#M0492S
OneTaq 2X master mix with standard buffer	NEB	Cat#M0482S

**Recombinant DNA**		

pEC86	Arslan et al. 1998, Mikrobiologisches Institut, Züric^58^	–
CymA-MtrCAB-pET30a(+)	Jensen et al., 2016, University of California, Berkeley^26^	–
CymA-MtrC-SpyTag-MtrAB-pET30a(+)	This study	–
CymA-MtrC-GrBP5-MtrAB-pET30a(+)	This study	–
pCD24r1	Dundas et al. University of Texas, Austin^36^	–
pCD24r1-SpyTag	This study	–
pCD24r1-GrBP5	This study	–
MtrCAB-pCD24r1	This study	–
MtrCAB-SpyTag-pCD24r1	This study	–
pCD24r6	Dundas et al. University of Texas, Austin^36^	–
pCD24r6-SpyTag	This study	–
pKEL10	This study	–
mCerulean3-SpyCatcher-pKEL10	This study	–
mKate2-SpyCatcher-pKEL10	This study	–

**Software and algorithms**		

AlphaFold 3	Google DeepMind	https://alphafoldserver.com/
ChimeraX 1.8	UC San Francisco	https://www.cgl.ucsf.edu/chimerax/download.html
AfterMath	Pine Research	https://pineresearch.com/downloads/
DropView 8000M	Metrohm	https://metrohm-dropsens.com/products/software/dropview-8400m-software/
Prism 10	GraphPad	https://www.graphpad.com/features
BioRender	BioRender	https://www.biorender.com/

**Other**		

PEEK/copper electrode holder	Xi’an Yima Opto-electrical Technology	–
Ag/AgCl reference electrode	Xi’an Yima Opto-electrical Technology	–
AvCarb G280A carbon felt	FuelCellStore	Cat#1595085
Aquivion E98-15s ion exchange membrane	FuelCellStore	Cat#72700014
MEC custom glassware and caps	Adams and Chittenden	–
Heated stirrer with temperature probe	Westlab	Cat#663-575
WaveNow wireless electrochemical workstation	WaveNow	Cat#AFTP4
μStat 8000 multichannel potentiostat	Metrohm	Cat#DRP-STAT8000
CLARIOstar Plus spectrophotometer	BMG Labtech	–
Concept C400 Anaerobic chamber	Baker Ruskinn	–
LSM 780 confocal microscope	Zeiss	–
Amicon Ultra centrifugal columns	Millipore	Cat#UFC903024
Sonifier SFX250 sonicator	Branson	–
Q3000T D Plus sputterer	Quorum	–
TM4000Plus electron microscope	Hitachi	–

### Experimental model and study participant details

#### Bacterial strains, culturing conditions, and growth medium

The *E. coli* NEB Turbo and *E. coli* BL21(DE3) strains were obtained from New England Biolabs (Table S2). The *S. oneidensis* MR-1 WT, Δ*mtrC*/Δ*mtrF*/Δ*omcA* (JG596) and JG1194 cell lines were obtained from Jeffrey Gralnick (University of Minnesota, Table S2). Cell strains were stored as glycerol stocks at −80°C. For protein expression, the *E. coli* and *S. oneidensis* strains were cultured in 2xYT media at 37°C or 30°C, respectively. During MEC experiments, the *S. oneidensis* strains were cultured in SBM. The induction of protein expression for the *E. coli* and *S. oneidensis* strains used 400 μM or 1 mM IPTG, respectively.

### Method details

#### Protein modeling and plasmid assembly

The engineered MtrC fusion proteins were modeled using the AlphaFold 3 server^35^ and visualized using UCSF ChimeraX software^59^ to determine designs that avoid disrupting protein folding. Subsequently, plasmids to express the modified MtrC proteins in *S. oneidensis* were created by Q5 PCR amplification of the backbone of existing MtrC expression plasmids (Table S3) using primers (Table S4) that generate a linear DNA fragment that begins and ends at the C-terminus of the *mtrC* gene, followed by Gibson assembly with gBlocks (IDT) encoding either the SpyTag or GrBP5 domain, as well as the linker sequence and plasmid homology sequences. The MtrC expression strains of *S. oneidensis* were created by electroporating plasmids that encode MtrC or MtrC-SpyTag into *S. oneidensis* Δ*mtrC*/Δ*mtrF*/Δ*omcA*, or plasmids encoding MtrCAB or MtrCAB-SpyTag into *S. oneidensis* JG1194 and grown on lysogeny broth (LB)-agar plates containing kanamycin.^60^ The *S. oneidensis* MtrC expression plasmids consisted of a Ribozyme J modulated, IPTG inducible PtacsymO promoter alongside a weak RBS (pCD24r1) or strong RBS (pCD24r6). The CymA-MtrCAB expression strains of *E. coli* were created by transforming an assembled CymA-MtrCAB or CymA-MtrCAB-SpyTag plasmids into *E. coli* BL21(DE3) cells containing the pEC86 heme maturation vector and grown on kanamycin/chloramphenicol media.^58^ The CymA-MtrCAB expression strains in *E. coli* consisted of a T7 inducible promoter and operator within a pET30a(+) plasmid backbone. Plasmids that encode the fluorescent proteins fused to SpyCatcher were assembled using Golden Gate Assembly to insert gBlocks gene fragments (IDT) into a pKEL10 T7 recombinant expression plasmid backbone, followed by transformation into *E. coli* BL21(DE3) cells. Plasmid sequences were verified by OneTaq colony PCR, followed by Sanger sequencing (Ramaciotti Center for Genomics). A summary of plasmids and bacterial strains used in this study is shown in Tables S1 and S2, and complete plasmid sequences are available upon request.

#### Protein expression

The MtrCAB proteins were expressed in *E. coli* grown in 2x-yeast-tryptone (2xYT) media containing 50 μg/mL kanamycin and 25 μg/mL chloramphenicol, and the mCerulean3-SpyCatcher and mKate2-SpyCatcher proteins were expressed in *E. coli* grown in lysogeny broth (LB) containing 100 μg/mL of ampicillin. The *E. coli* cultures were grown at 37°C with shaking to an optical density (OD) of 0.5 at 600 nm, and protein expression induced at 18°C for 15 h with the addition of IPTG to a final concentration of 400 μM, unless otherwise described. Recombinant expression of modified and unmodified MtrC in *S. oneidensis* was performed similar to the MtrCAB expression in *E. coli* with the exception of an initial growth at 30°C followed by expression induction with the addition of IPTG to a final concentration of 1 mM.

#### Protein purification

The recombinantly expressed mCerulean3-SpyCatcher and mKate2-SpyCatcher proteins were extracted from *E. coli* by suspending the cells in binding buffer (20 mM sodium phosphate buffer, 500 mM NaCl, 10 mM imidazole, pH 8) and lysing via sonication (Sonifier SFX250, Branson) for 8 min, pulsing 2 s on and 2 s off. The lysate was centrifuged twice at 10,000 *g* for 30 min, and the supernatant syringe filtered and flowed through an Ni-NTA column (Thermo Fisher Scientific) equilibrated with binding buffer. Protein was purified using an elution gradient containing increasing concentrations of imidazole up to 40 mM in binding buffer and eluted with 240 mM imidazole. The eluted pure protein was concentrated and buffer exchanged into phosphate buffered saline (PBS; 10 mM Na_2_HPO_4_, 1.8 mM KH_2_PO_4_, 2.7 mM KCl, 137 mM NaCl, pH 7.4) using Amicon Ultra centrifugal columns (Merck). Protein concentration was determined by Bradford assay (Bio-Rad) and the size and purity of the proteins confirmed by sodium dodecyl sulfate polyacrylamide gel electrophoresis (SDS-PAGE). Purified protein was then mixed in a 1:1 ratio with sterile 50% glycerol, flash frozen in liquid nitrogen, and stored at −80°C.

#### Quantification and imaging of bioconjugation

The binding of the fluorescent mCerulean3-SpyCatcher and mKate2-SpyCatcher proteins to the engineered or wild-type MtrC on the surface of *E. coli* or *S. oneidensis* was measured by spectrophotometer and fluorescence microscopy. The different cell strains were resuspended in PBS, normalized to an OD of 1.5 at 600 nm and incubated on a rotor at room temperature overnight with addition of either a fluorophore-SpyCatcher in PBS at a final concentration of 1 μM, or only PBS as a cell background fluorescence control. Subsequently, the cells were washed three times by centrifugation and resuspension in 1 mL PBS, followed by resuspension in 100 μL PBS and transfer to black 96-well microtiter plates. The fluorescence of the cells bioconjugated to mCerulean3 was analyzed using a spectrophotometer (CLARIOstar plus, BMG) with an excitation at 433 nm and emission at 500 nm, and corrected by subtracting the PBS background control. Similarly, the fluorescence of cells bioconjugated to mKate2 was analyzed using excitation at 588 nm and emission at 633 nm and corrected against the PBS background control. The mCerulean3 fluorescence of the bioconjugated and control cells was imaged by fluorescence microscopy by transferring 10 μL of cells on a microscope slide, covered with a coverslip, and sealed with clear nail polish. Cells were imaged using a confocal microscope (Zeiss LSM 780) with 200× magnification, with the mCerulean3 excited with a 405 nm light source, and cell morphology imaged using white light bright field excitation.

#### Ferrozine assay

The reduction of iron(III) by the engineered and wild-type *S. oneidensis* strains was performed using a ferrozine assay. Overnight cultures of *S. oneidensis* were prepared in sealed falcon tubes containing *Shewanella* Basal Media (SBM) supplemented with the electron donor sodium lactate (20 mM), electron acceptor sodium fumarate (40 mM), and kanamycin (50 μg/mL). The SBM contained 0.46 g NaCl, 0.225 g K_2_HPO_4_, 0.225 g KH_2_PO_4_, 0.117 g MgSO_4_·7H_2_O, 0.225 g of (NH_4_)_2_SO_4_, 0.5 g casamino acids (0.05%), 10 mL trace mineral supplement (ATCC) (1% v/v), 10 mM HEPES, pH 7.2 to a final volume of 1 L in dH_2_O, and filter sterilized. The ferrozine assay was performed as described previously.^36^ Briefly, anaerobic media was prepared by bubbling nitrogen through a 0.22 μm filter for 30 min while stirring. In an anaerobic chamber (Concept C400, Baker Ruskinn), ferrozine reactions were prepared in a transparent 96-well microplate (Sarstedt) by mixing 99 μL anaerobic SBM containing sodium lactate (20 mM), ferrozine (1 mg/mL), iron(III) citrate (2 mM), IPTG (1 mM) and kanamycin as required (50 μg/mL) with 1 μL of the relevant overnight *S. oneidensis* cell culture. In addition, a standard curve of SBM containing a linear gradient of iron(III)-iron(II) concentrations was used for quantification. The plate was incubated anaerobically at RT for 12 h, samples diluted by half in dH_2_O and plate absorbance measured at 562 nm using a spectrophotometer (CLARIOstar, BMG).

#### Methyl orange assay

Electron transfer by engineered *E. coli* strains was measured using the colourimetric methyl orange assay, as described previously.^27^ Briefly, overnight seed cultures were prepared in 5 mL 2xYT media containing chloramphenicol (25 μg/mL) and kanamycin (50 μg/mL) as required, overnight at 37°C, 200 rpm. Subsequently, 40 mL expression cultures of 2xYT supplemented with 1 mM 5-aminolevulinic acid, as well as chloramphenicol (25 μg/mL) and kanamycin (50 μg/mL) as required, were inoculated with 400 μL of seed culture and grown aerobically to an OD600 of 0.5 at 37°C, 200 rpm, then induced with 10 μM IPTG. Induced cultures were grown overnight aerobically at 30°C, 200 rpm, harvested at 3000 *g*, 4°C, 15 min, and washed in 10 mL M9-Glycerol. The M9-glycerol contained 1X M9 salts, 1 mM MgSO_4_, 100 μM CaCl_2_, 0.2% casamino acids and 50 mM glycerol, at pH 7.2. Cells were then gently resuspended in 10 mL M9-glycerol containing 200 μM methyl orange, transferred to a 15 mL tube and sealed tightly for semi-anaerobic growth. After 24 h, the culture was clarified at 21,000 *g*, 3 min, and the absorbance of 200 μL of supernatant measured at 465 nm using a spectrophotometer (CLARIOstar, BMG). Absorbance measurements were converted to a change in methyl orange concentration by comparison to a serial dilution standard curve.

#### MEC assembly and chronoamperometry

Chronoamperometry was performed in a two-chamber MEC in an H-configuration, with two borosilicate media bottles (Adams & Chittenden) connected through a flange that contains Aquivion E98-15s ion exchange membrane (FuelCellStore). The working and counter electrodes were AvCarb G280A carbon felt (4.50 cm × 3.50 cm x 0.28 cm, 35.98 cm^2^ geometric surface area, FuelCellStore). The RBS strength test (Figure S2) used a smaller electrode (2 cm × 2 cm x 0.28 cm, 10.24 cm^2^), but was set up identically otherwise. The assembled MEC was connected to the potentiostat (WaveNow, Pine Research, or DropSens, Metrohm) using the provided crocodile clips. A detailed image of the assembled MEC is shown in the supplemental information (Figure S4). Deoxygenated SBM supplemented with sodium lactate (20 mM), kanamycin (50 μg/mL) and IPTG (1 mM) was prepared by bubbling sterile nitrogen for 30 min while stirring. MECs were assembled in an anaerobic chamber at 0% oxygen with 100 mL of deoxygenated SBM added to the working chamber. Overnight cultures of *S. oneidensis* strains were prepared in SBM supplemented with sodium lactate (20 mM) and sodium fumarate (40 mM), grown overnight at 30°C, normalized to an OD600 of 1.5 and 1 mL added to the working chamber. The carbon felt working electrode was fixed in place using a PEEK/copper sample clamp (Xi’an Yima Opto-electrical Technology). The working electrode sample clamp and Ag/AgCl reference electrode (Xi’an Yima Opto-electrical Technology) were inserted into the working chamber and an airtight seal formed using silicone sealing rings. The assembled airtight MEC was transferred from the anaerobic chamber to a heated stirrer set to 30°C, 200 rpm with magnetic stir bar, and the temperature probe was taped to the side of the working chamber. The counter chamber was filled with 100 mL of dH_2_O and a side port was left open for oxygen transfer and the assembled MEC was then ready for chronoamperometry (Figure S4). A chronoamperometry protocol for *S. oneidensis* (AfterMath, Pine Research, or DropView 8400M, Metrohm) was performed using a potentiostat (WaveNow, Pine Research, or DropSens, Metrohm) with an applied potential of 0 V vs. Ag/AgCl reference (205 mV vs. SHE) for 48 h, with the current measured each minute.

#### Cyclic voltammetry

Each bacterial strain was prepared for CV by growing cells under 0 V (205 mV vs. SHE) applied potential for 24 h using the MEC setup described above to develop a representative biofilm on the graphite felt electrode and induce recombinant protein production. To ensure substrate turnover, fresh deoxygenated SBM, containing antibiotics as required, was added under anaerobic conditions and chronoamperometry at 0 V (205 mV vs. SHE) applied potential for a further 6 h.^61^ Using the same setup used for chronoamperometry (Figure S4), CV was performed for three cycles between −0.7 V (−0.495 V vs. SHE) and 0.3 V (505 V vs. SHE), with a scan rate of 1 mV/s.

#### Graphite-cell binding assay

Overnight seed cultures were prepared as described above using either *S. oneidensis* Δ*mtrC*/Δ*mtrF*/Δ*omcA* cells expressing MtrC or MtrC-GrBP5, or *E. coli* BL21(DE3) cells expressing CymA-MtrCAB or CymA-MtrCAB-GrBP5. The following day, seed cultures were used to inoculate 100 mL induction cultures in a baffled flask containing a sterile square of 2 cm × 2 cm x 0.28 cm carbon felt (AvCarb G280A, FuelCellStore) submerged in 2xYT media containing antibiotics as appropriate. Cell cultures were grown at 30°C for *S. oneidensis*, or 37°C for *E. coli*, with shaking at 200 rpm. Expression was induced with 1 mM IPTG for *S. oneidensis* or 400 μM IPTG for *E. coli*, and cultures were incubated overnight. After overnight induction of protein expression, the graphite felt was washed three times in 15 mL PBS for 1 h with gentle shaking. Circles of carbon felt with a diameter of 6.5 mm were isolated with a hole punch and incubated in 50 μL of 0.5% w/v SDS, 50 mM Tris-HCl, pH 8 for 20 min at RT. The samples were centrifuged at 13,000 *g* for 5 min to pellet felt fibers and the clarified supernatant transferred to a fresh tube and diluted 1 in 5 with dH_2_O. The protein concentration of the samples was quantified by Bradford assay (Bio-Rad) against a standard curve of BSA (Bio-Rad). The total quantity of protein from lysed cells that were bound to carbon felt circle was calculated based on a total projected surface area of 0.6637 cm^2^ (holepunch diameter 0.65 cm).

#### SEM of graphite felt

The attachment of *E. coli* or *S. oneidensis* cells to graphite electrodes was imaged by SEM. Graphite felt was prepared using the graphite-cell binding assay, or removed from an MEC after 11.5 h of operation. Graphite felt was washed 3 times for 1 h in PBS with gentle shaking, and dehydrated by incubation for 10 min in increasing concentrations of ethanol (50%, 60%, 70%, 80%, 90%, 100%), and dried for 20 min at 37°C. Hole-punched circles of dried graphite felt were coated with a gold layer to a depth of 2 nm using a sputterer (Q300T D Plus, Quorum) and mounted on an SEM holder with conductive carbon tape. Images were acquired using an electron microscope (TM4000Plus, Hitachi).

### Quantification and statistical analysis

The statistical details of the experiments are presented in Figures 2, 3, 4, and 5. Statistical significance was calculated using GraphPad Prism 10. A one-tailed parametric *t* test was used when *a priori* directional hypothesis of increased response was specified; otherwise, two-tailed parametric t-tests were used to assess differences.
