# Supplementary material for: Engineering electron conduits in bacteria for selective biointerfacing and enhanced energy transfer
Source: iScience. 2026 Jan 28;29(2):114805. doi: 10.1016/j.isci.2026.114805 (PMC12915252; doi:10.1016/j.isci.2026.114805)
Supplement: Document S1. Figures S1–S5 and Tables S1–S3 [file mmc1.pdf]

iScience, Volume *29*

Supplemental information

Engineering electron conduits in bacteria for selective biointerfacing and enhanced energy transfer

Alexander R. Kelly, Lorenzo Travaglini, and Dominic J. Glover

Contents

Figure S1......Predicted AlphaFold 3 structural protein model of MtrCAB-SpyTag and MtrCAB-GrBP5

Figure S2......MEC chronoamperometry of *S. oneidensis* Δ*mtrC*/Δ*mtrF*/Δ*omcA* recombinantly expressing MtrC under the control of a weak (pCD24r1) or strong (pCD24r6) RBS

Figure S3......Optimization of MtrCAB-SpyTag expression in *S. oneidensis* and fluorescence bioconjugation

Figure S4......Microbial electrolysis cell setup and software conditions

Figure S5......SEM of graphite felt electrodes after 11.5 h of MEC operation

Table S1......Fusion protein sequences used in this study

Table S2......Bacterial host strains used in this study

Table S3......Plasmid DNAs used in this study

Table S4......Oligonucleotides used in this study

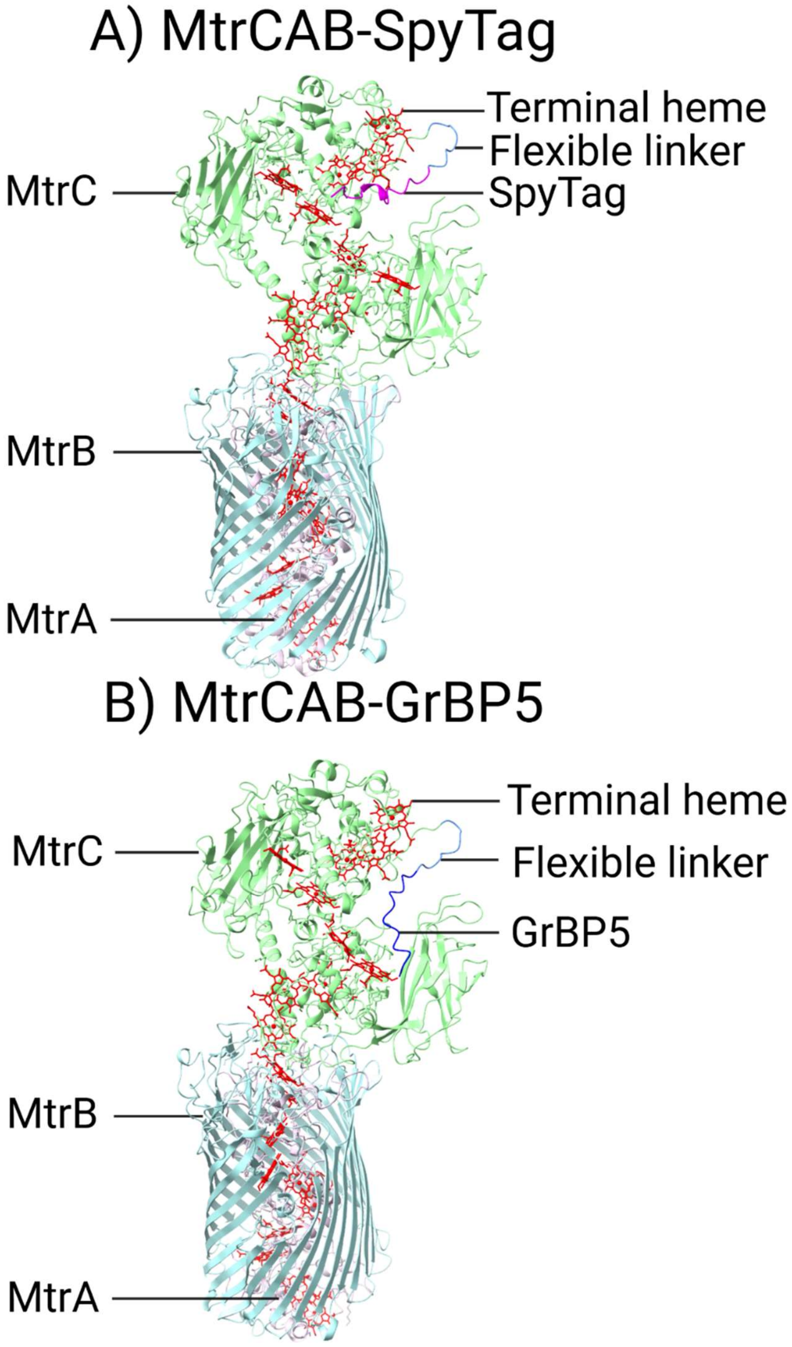

Figure S1. Predicted AlphaFold 3 structural protein model of MtrCAB-SpyTag and MtrCAB-GrBP5. The MtrCAB complex is fused to either a SpyTag or GrBP5 domain through a GSGESGSG flexible linker (single letter amino acid code). The SpyTag and GrBP5 domains are exposed to the extracellular space and are in close proximity to the most distal heme group of MtrC. Heme molecules are shown in red.

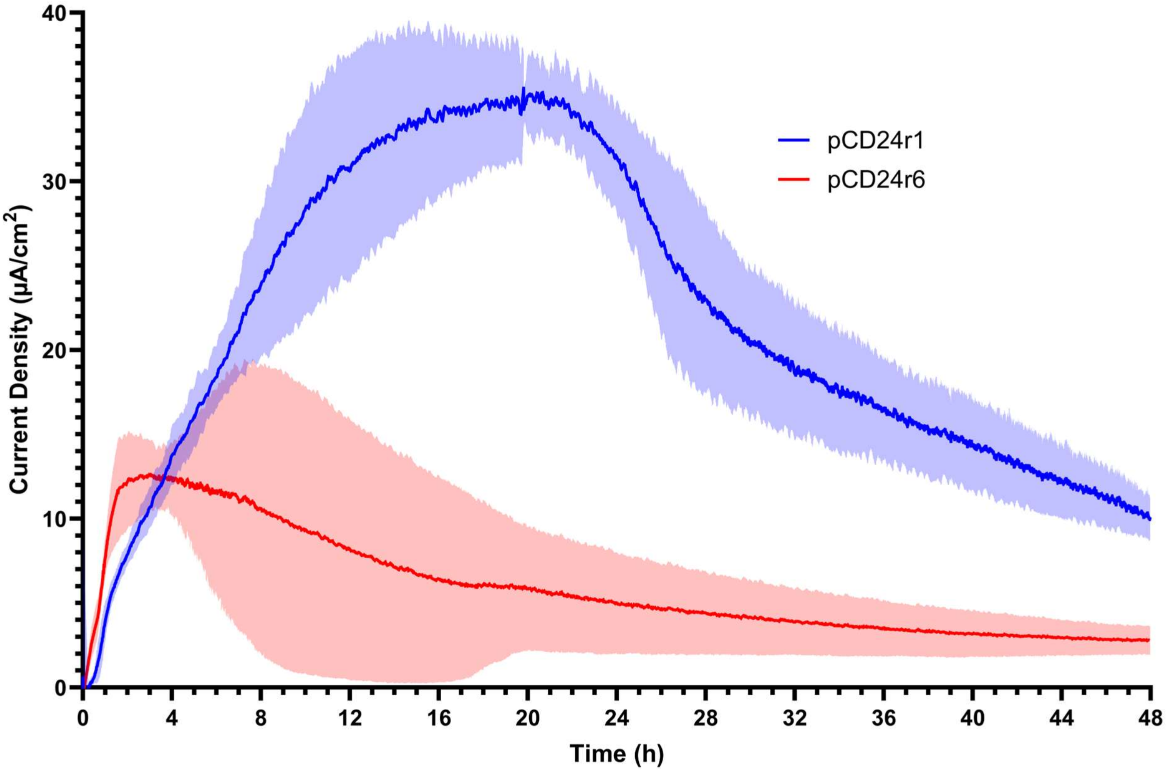

Figure S2. MEC chronoamperometry of *S. oneidensis* Δ*mtrC*/Δ*mtrF*/Δ*omcA* recombinantly expressing MtrC under the control of a weak (pCD24r1) or strong (pCD24r6) RBS. The cell strains were grown in MECs on 2 x 2 cm carbon felt working electrodes. Expression of MtrC under the control of a strong RBS resulted in initially faster production of current, which tapered off earlier, resulting in a lower overall maximum current density (μA/cm^2). The data represents the average current density of three separate biological repeats. Standard deviation shown as shaded area.

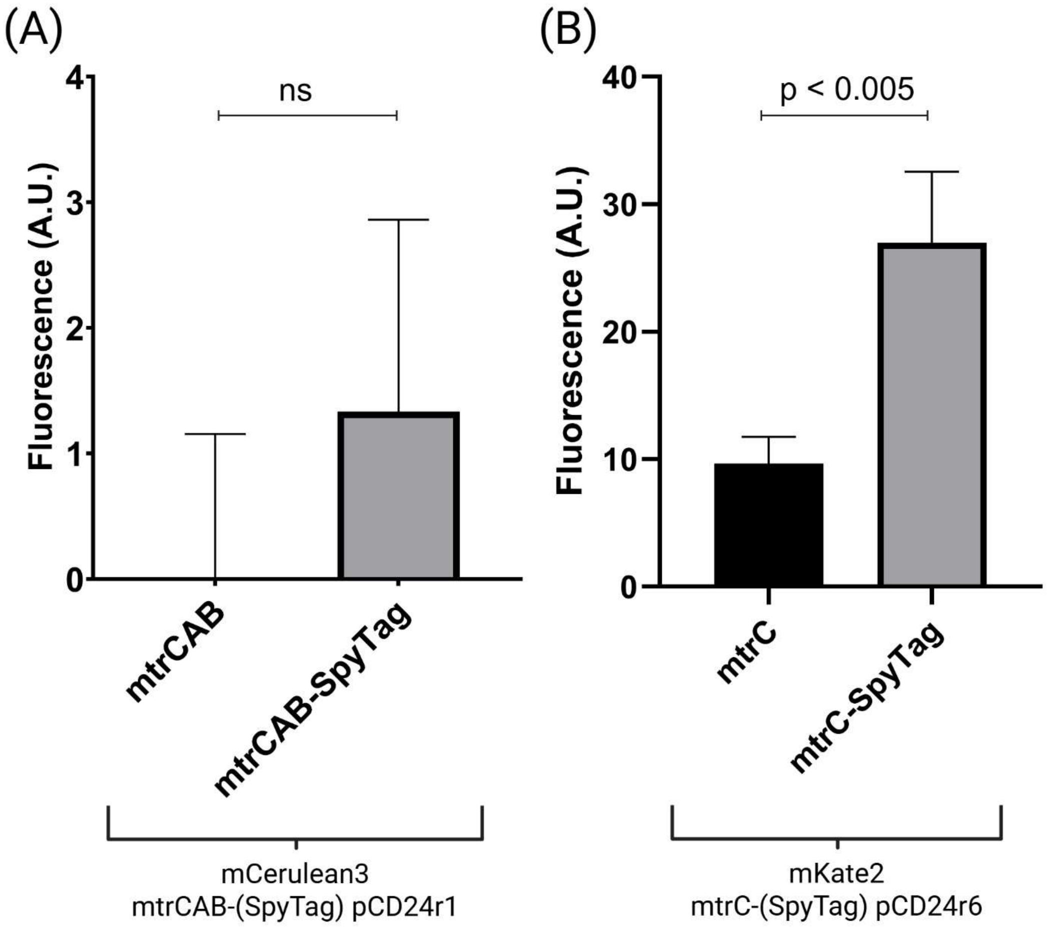

Figure S3. Optimization of MtrCAB-SpyTag expression in *S. oneidensis* and fluorescence bioconjugation. (A) Relative fluorescence of *S. oneidensis* JG1194 (Δ*mtrC*, *omcA*, Δ*mtrF*, Δ*mtrA*, Δ*mtrD*, Δ*dmsE*, Δ*so4360*, Δ*cctA* and Δ*recA*) cells recombinantly expressing MtrCAB or MtrC-SpyTag-MtrAB under the control of a weak pCD24r1 RBS after exposure to mCerulean3-SpyCatcher. Error bars represent standard deviation between experiments (n=3, significance calculated using a parametric, unpaired, one-tailed t-test). (B) Fluorescence of cells expressing MtrC or MtrC-SpyTag controlled by a strong RBS after reaction with mKate2-SpyCatcher. Error bars represent standard deviation between experiments (n=3, significance calculated using a parametric, unpaired, one-tailed t-test).

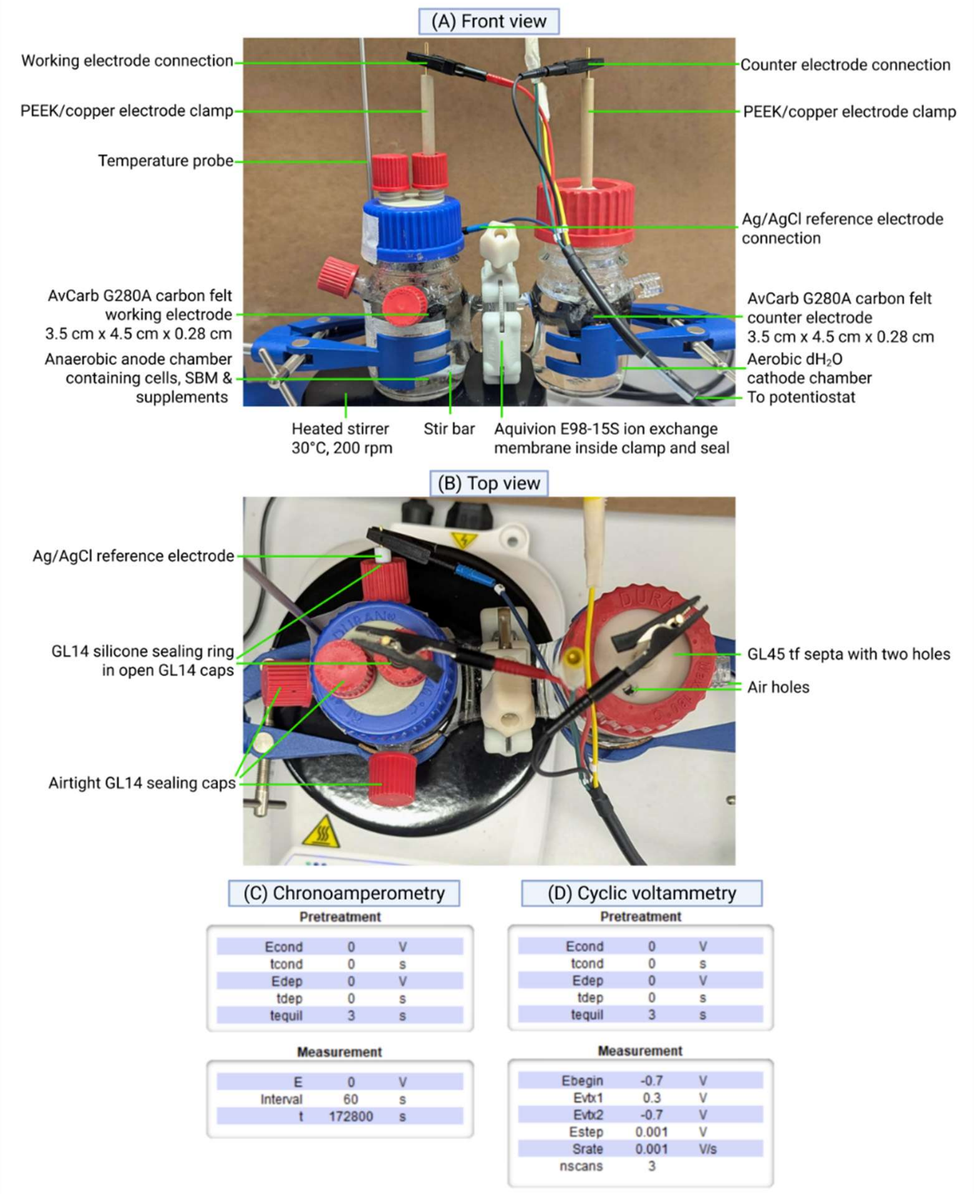

Figure S4. Microbial electrolysis cell (MEC) setup and potentiostat software operation conditions. (A) Front view of the MEC setup during operation. The left anaerobic anode chamber contains the cell culture of SBM +/- IPTG/kanamycin, the working electrode and the reference electrode. The aerobic chamber (right) contains distilled H_2O and the counter electrode. Electrodes are connected to the potentiostat using PEEK/copper sample clamps with a screw to hold the carbon felt firmly in place, linked to the potentiostat by crocodile clamps. (B) Top view of the MEC during operation. The Ag/AgCl reference electrode is held in place using an airtight silicone sealing ring and attached by a crocodile clip to the potentiostat. The ventilation holes in the top and side of the aerobic counter chamber are visible. (C) DropView 8000M chronoamperometry procedure for 48 h at 0V (vs. Ag/AgCl), measuring once per min. (D) DropView 8000M cyclic voltammetry procedure for 3 cycles of CV between –0.7 V and 0.3 V (vs. Ag/AgCl) with a scan rate of 1 mV/s.

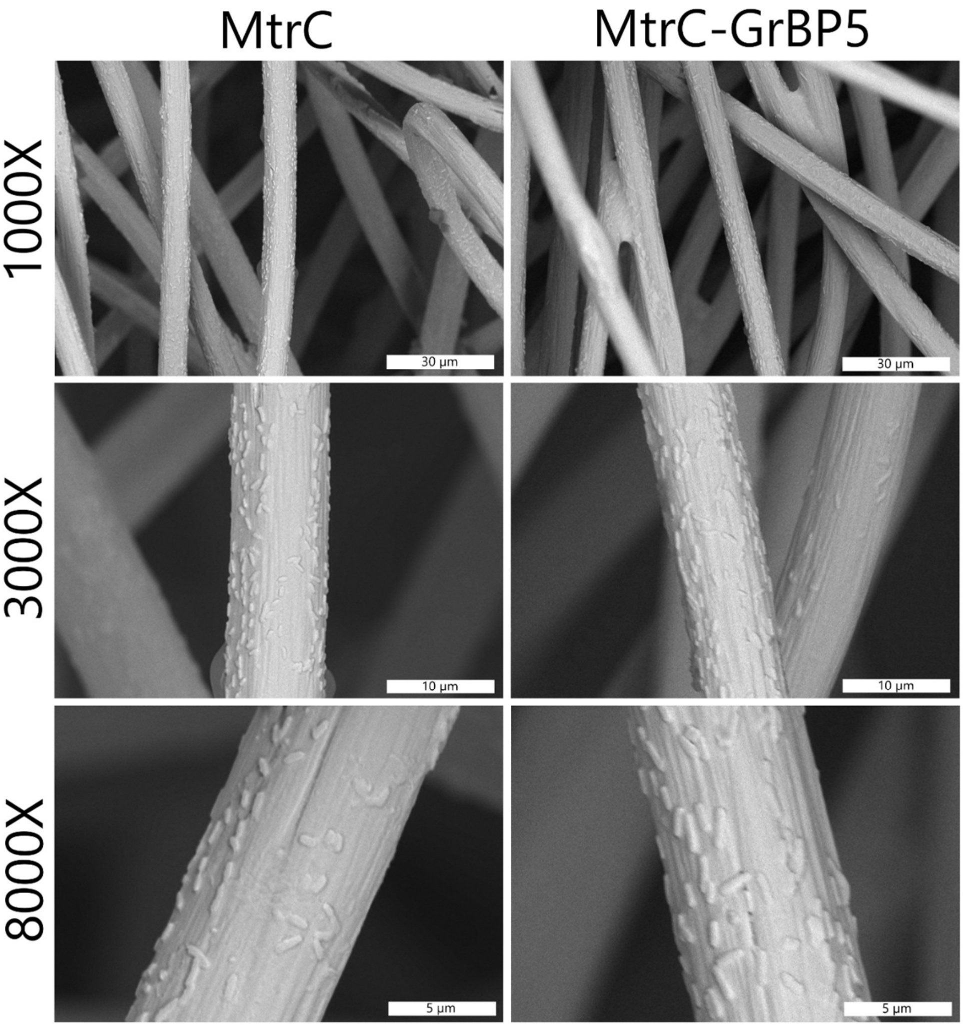

Figure S5. SEM of graphite felt electrodes after 11.5 h of MEC operation. SEM images of graphite felt at varying magnification after incubation for 11.5 h in an MEC with *S. oneidensis* Δ*mtrC*/Δ*mtrF*/Δ*omcA* cells expressing MtrC or MtrC-SpyTag. Both modified variants of *S. oneidensis* appeared to have a healthy population of adhered cells.

Table S1. Fusion protein sequences used in this study.

Proteins expressed	Features	Protein Sequence
mCerulean3-SpyCatcher	1. His-Tag<br>2. GSG linker<br>3. mCerulean3<br>4. GSG linker<br>5. SpyCatcher	MHHHHHHGSGVSKGEELFTGVVPILVELDGDVNGHKFSVSG<br>EGEGDATYGKLTLKFICTTGKLPVPWPTLVTTLSWGVQCFA<br>RYPDHMKQHDFFKSAMPEGYVQERTIFFKDDGNYKTRAEVK<br>FEGDTLVNRIELKGIDFKEDGNILGHKLEYNAIHGNVYITA<br>DKQKNGIKANFGLNCNIEDGSVQLADHYQQNTPIGDGPVLL<br>PDNHYLSTQSKLSKDPNEKRDHMVLLEFVTAAGITLGMDEL<br>YKGSGVDTLSGLSSEQGQSGDMTIEEDSATHIKFSKRDEDG<br>KELAGATMELRDSSGKTISTWISDGQVKDFYLYPGKYTFVE<br>TAAPDGYEVATAITFTVNEQGQVTVNGKATKGDAHI
mKate2-SpyCatcher	1. His-Tag<br>2. GSG linker<br>3. mKate2<br>4. GSG linker<br>5. SpyCatcher	MHHHHHHGSGVSELIKENMHMKLYMEGTVNNHHFKCTSEGE<br>GKPYEGTQTMRIKAVEGGPLPFAFDILATSFMYGSKTFINH<br>TQGIPDFFKQSFPEGFTWERVTTYEDGGVLTATQDTSLQDG<br>CLIYNVKIRGVNFPSNGPVMQKKTLGWEASTETLYPADGGL<br>EGRADMALKLVGGGHLICNLKTTYRSKKPAKNLKMPGVYYV<br>DRRLERIKEADKETYVEQHEVAVARYCDLPSKLGHRGSGVD<br>TLSGLSSEQGQSGDMTIEEDSATHIKFSKRDEDGKELAGAT<br>MELRDSSGKTISTWISDGQVKDFYLYPGKYTFVETAAPDGY<br>EVATAITFTVNEQGQVTVNGKATKGDAHI
MtrC-SpyTag	1. MtrC<br>2. GSGESGSG linker<br>3. SpyTag	MMNAQKSKIALLLAASAVTMALTGCGGSDGNNGNDGSDGGE<br>PAGSIQTLNLDITKVSYENGAPMVTVFATNEADMPVIGLAN<br>LEIKKALQLIPEGATGPGNSANWQGLGSSKSYVDNKNGSYT<br>FKFDAFDSNKVFNAQLTQRFNVVSAAGKLADGTTVPVAEMV<br>EDFDGQGNAPQYTKNIVSHEVCASCHVEGEKIYHQATEVET<br>CISCHTQEFADGRGKPHVAFSHLIHNVHNANKAWGKDNKIP<br>TVAQNIVQDNCQVCHVESDMLTEAKNWSRIPTMEVCSSCHV<br>DIDFAAGKGHSQQLDNSNCIACHNSDWTAELHTAKTTATKN<br>LINQYGIETTSTINTETKAATISVQVVDANGTAVDLKTILP<br>KVQRLEIITNVGPNNATLGYSGKDSIFAIKNGALDPKATIN<br>DAGKLVYTTTKDLKLGQNGADSDTAFSFVGWSMCSSEGKFV<br>DCADPAFDGVDVTKYTGMKADLAFATLSGKAPSTRHVDSVN<br>MTACANCHTAEFEIHKGKQHAGFVMTEQLSHTQDANGKAIV<br>GLDACVTCHTPDGTYSFANRGALELKLHKKHVEDAYGLIGG<br>NCASCHSDFNLESFKKKGALNTAAAADKTGLYSTPITATCT<br>TCHTVGSQYMVHTKETLESFGAVVDGTKDDATSAAQSETCF<br>YCHTPTVADHTKVKMGSGESGSGAHIVMVDAYKPTK
MtrC-GrBP5	1. MtrC<br>2. GSGESGSG linker<br>3. GrBP5	MMNAQKSKIALLLAASAVTMALTGCGGSDGNNGNDGSDGGE<br>PAGSIQTLNLDITKVSYENGAPMVTVFATNEADMPVIGLAN<br>LEIKKALQLIPEGATGPGNSANWQGLGSSKSYVDNKNGSYT<br>FKFDAFDSNKVFNAQLTQRFNVVSAAGKLADGTTVPVAEMV<br>EDFDGQGNAPQYTKNIVSHEVCASCHVEGEKIYHQATEVET<br>CISCHTQEFADGRGKPHVAFSHLIHNVHNANKAWGKDNKIP<br>TVAQNIVQDNCQVCHVESDMLTEAKNWSRIPTMEVCSSCHV<br>DIDFAAGKGHSQQLDNSNCIACHNSDWTAELHTAKTTATKN<br>LINQYGIETTSTINTETKAATISVQVVDANGTAVDLKTILP

KVQRLEIITNVGPNNATLGYSGKDSIFAIKNGALDPKATIN
DAGKLVYTTTKDLKLGQNGADSDTAFSFVGWSMCSSEGKFV
DCADPAFDGVDVTKYTGMKADLAFATLSGKAPSTRHVDSVN
MTACANCHTAEFEIHKGKQHAGFVMTEQLSHTQDANGKAIV
GLDACVTCHTPDGTYSFANRGALELKLHKKHVEDAYGLIGG
NCASCHSDFNLESFKKKGALNTAAAADKTGLYSTPITATCT
TCHTVGSQYMVHTKETLESFGAVVDGTKDDATSAAQSETCF
YCHTPTVADHTKVKMGSGESGSGIMVTESSDYSSY

Table S2. Bacterial host strains used in this study.

**Bacterial host**	**Function**	**Source**
E. coli NEB Turbo	Cloning strain	NEB
E. coli BL21(DE3)	Recombinant protein expression under the T7 operon	NEB
S. oneidensis MR-1 WT	Wildtype S. *oneidensis* MR-1	Myers and Nealson 1988, University of Wisconsin, Milwaukee[1]
S. oneidensis Δ*mtrC*/Δ*mtrF*/Δ*omcA* (JG596)	*S. oneidensis* strain with the genes for the outer membrane proteins *mtrC*, *mtrF* and *omcA* knocked out	Coursolle & Gralnick 2010, University of Minnesota, Twin Cities[2]
S. oneidensis JG1194	*S. oneidensis* strain with the genes for the proteins *mtrC*, *omcA*, *mtrF*, *mtrA*, *mtrD*, *dmsE*, *so4360*, *cctA* and *recA* knocked out	Coursolle & Gralnick 2012, University of Minnesota, Twin Cities[3]

Table S3. Plasmid DNAs used in this study.

**Plasmid**	**Encoded genes and intended host**	**Resistance**	**Source**
pEC86	C-type cytochrome maturation proteins ccmABCDEFGH constitutively expressed within a Tet-Off expression system.	Chloramphenicol	Arslan et al. 1998, Mikrobiologisches Institut, Zürich[4]

CymA-MtrCAB-pET30a(+)	*cymA*, *mtrC*, *mtrA* and *mtrB* genes held within a T7 operon in a pET30a(+) backbone. For expression in *E. coli*.<br><br>**Shorthand: MtrCAB**	Kanamycin	Jensen et al., 2016, University of California, Berkeley[5]
CymA-MtrC-SpyTag-MtrAB-pET30a(+)	*cymA*, *mtrC* with a C-terminal fused *SpyTag*, *mtrA* and *mtrB* genes held within a T7 operon in a pET30a(+) backbone. For expression in *E. coli*.<br><br>**Shorthand: MtrCAB-SpyTag**	Kanamycin	This study
CymA-MtrC-GrBP5-MtrAB-pET30a(+)	*cymA*, *mtrC* with a C-terminal fused *grBP5* graphite binding domain, *mtrA* and *mtrB* genes held within a T7 operon in a pET30a(+) backbone. For expression in *E. coli*.<br><br>**Shorthand: MtrCAB-GrBP5**	Kanamycin	This study
pCD24r1	*mtrC* gene controlled by a Ribozyme J modulated, IPTG inducible $P_{tacsymO}$ promoter in a pCD backbone with a weak RBS. For expression in *S. oneidensis*.<br><br>**Shorthand: MtrC (weak RBS)**	Kanamycin	Dundas et al. University of Texas, Austin[6]
pCD24r1-SpyTag	*mtrC* with a C-terminal fused *spyTag* gene controlled by a Ribozyme J modulated, IPTG inducible $P_{tacsymO}$ promoter in a pCD backbone with a weak RBS. For expression in *S. oneidensis*.<br><br>**Shorthand: MtrC-SpyTag**	Kanamycin	This study
pCD24r1-GrBP5	*mtrC* with a C-terminal fused *grBP5* graphite binding gene controlled by a Ribozyme J modulated, IPTG inducible $P_{tacsymO}$ promoter in a pCD backbone. For expression in *S. oneidensis*.<br><br>**Shorthand: MtrC-GrBP5**	Kanamycin	This study
MtrCAB-pCD24r1	*mtrC*, *mtrA* and *mtrB* genes controlled by a Ribozyme J modulated, IPTG inducible $P_{tacsymO}$ promoter in a pCD backbone.	Kanamycin	This study

MtrCAB-SpyTag - pCD24r1	*mtrC* with a C-terminal fused *SpyTag*, *mtrA* and *mtrB* genes controlled by a Ribozyme J modulated, IPTG inducible $P_{tacsymO}$ promoter in a pCD backbone.	Kanamycin	This study
pCD24r6	*mtrC* gene controlled by a Ribozyme J modulated, IPTG inducible $P_{tacsymO}$ promoter in a pCD backbone with a strong RBS. For expression in *S. oneidensis*. **Shorthand: MtrC (strong RBS)**	Kanamycin	Dundas et al. 2020, University of Texas, Austin[6]
pCD24r6-SpyTag	*mtrC* with a C-terminal fused *SpyTag* gene controlled by a Ribozyme J modulated, IPTG inducible $P_{tacsymO}$ promoter in a pCD backbone with a strong RBS. For expression in *S. oneidensis*. **Shorthand: MtrC-SpyTag (strong RBS)**	Kanamycin	This study
pKEL10	Novel backbone designed for easy Golden Gate assembly for the expression of recombinant proteins under a T7 operon. Contains a blue-white colony screen which functions via a nested constitutive promoter encoding a blue chromoprotein.	Ampicillin	This study
mCerulean3-SpyCatcher-pKEL10	His-tagged *mCerulean3* with a C-terminal fused *SpyCatcher* bioconjugation domain held within a T7 operon in a pKEL10 backbone.	Ampicillin	This study
mKate2-SpyCatcher-pKEL10	His-Tagged *mKate2* fluorescent protein with a C-terminal fused *SpyCatcher* bioconjugation domain held within a T7 operon in a pKEL10 backbone.	Ampicillin	This study

Table S4. Oligonucleotides used in this study.

Oligonucleotide	Sequence	Function	Source
Fwd-Gibson-pCD24r1	gtcacactggctcaccttcgg	pCD24r1/pCD24r6 backbone amplification for Gibson assembly with SpyTag and GrBP5<br>**Final products: pCD24r1-SpyTag, pCD24r1-GrBP5, pCD24r1-GrBP5**	This study
Rvs-Gibson-pCD24r1	tgttggggtatggcagtagaaacagg	pCD24r1/pCD24r6 backbone amplification for Gibson assembly with SpyTag and GrBP5<br>**Final products: pCD24r1-SpyTag, pCD24r1-GrBP5, pCD24r1-GrBP5**	This study
pCD24r1 Fwd Gibson MtrCAB homology	gcatgagttacaaactctaaTAATCTAGACCAGGCATCAAATAAAACG	Amplification of pCD24r1 backbone for assembly with MtrCAB/MtrCAB-SpyTag<br>**Final products: pCD24r1-MtrCAB, pCD24r1-MtrCAB-SpyTag**	This study
pCD24r1 Rvs Gibson MtrCAB homology	tttgatttttgtgcgttcatATCGCACTGCTGTTTTTCCC	Amplification of pCD24r1 backbone for assembly with MtrCAB/MtrCAB-SpyTag<br>**Final products: pCD24r1-MtrCAB, pCD24r1-MtrCAB-SpyTag**	This study
MtrCAB Fwd Gibson pCD24r1 Homology	GGGAAAAACAGCAGTGCGATatgaacgcacaaaaatcaaaaatcg	Amplification of MtrCAB/MtrCAB-SpyTag for Gibson assembly with pCD24r1<br>**Final products: pCD24r1-MtrCAB, pCD24r1-MtrCAB-SpyTag**	This study
MtrCAB Rvs Gibson pCD24r1 Homology	TTGATGCCTGGTCTAGATTAttagagtttgtaactcatgctcagc	Amplification of MtrCAB/MtrCAB-SpyTag for Gibson assembly with pCD24r1<br>**Final products: pCD24r1-MtrCAB, pCD24r1-MtrCAB-SpyTag**	This study
Fwd-SpyTag-Backbone-Gibson	tgttggggtatggcagtagaaacagg	CymA-MtrCAB-pET30a(+) backbone amplification for SpyTag and GrBP5 insertion<br>**Final products: CymA-MtrC-SpyTag-MtrAB-pET30a(+), CymA-MtrC-GrBP5-MtrAB-pET30a(+)**	This study

Rvs-SpyTag-Backbone-Gibson	ctcccccctttcttgaattttgttgggac	CymA-MtrCAB-pET30a(+) backbone amplification for SpyTag and GrBP5 insertion<br>**Final products: CymA-MtrC-SpyTag-MtrAB-pET30a(+), CymA-MtrC-GrBP5-MtrAB-pET30a(+)**	This study
MtrB External Sequencing	gagaactataaggacaatgacg	Colony PCR/sequencing of plasmids containing MtrB	This study
MtrC Internal Sequencing	tctatattctacgccgatcac	Colony PCR/sequencing of plasmids containing MtrC	This study
Ptacsym0 Sequencing	CGGCTCGTATAATGTGTG	Colony PCR/sequencing of plasmids containing a PtacsymO promoter	This study
OriTrp4 Sequencing	ACAGATGAGGGCAAGC	Colony PCR/sequencing of plasmids containing an OriTrp4 origin of replication	This study
GrBP5 Sequencing	TGACCGAATCGAGCG	Colony PCR/sequencing of plasmids containing a GrBP5 binding domain	This study

Supplemental Information References

1. Myers, C.R., and Nealson, K.H. (1988). Bacterial manganese reduction and growth with manganese oxide as the sole electron acceptor. Science *240*, 1319–1321. https://doi.org/10.1126/science.240.4857.1319.

2. Coursolle, D., and Gralnick, J.A. (2010). Modularity of the Mtr respiratory pathway of *Shewanella oneidensis* strain MR-1. Mol. Microbiol. *77(4)*, 995–1008. https://doi.org/10.1111/j.1365-2958.2010.07266.x.

3. Coursolle, D., and Gralnick, J.A. (2012). Reconstruction of extracellular respiratory pathways for Iron(III) reduction in *Shewanella oneidensis* strain MR-1. Front. Microbio. *3*. https://doi.org/10.3389/fmicb.2012.00056.

4. Arslan, E., Schulz, H., Zufferey, R., Künzler, P., and Thöny-Meyer, L. (1998). Overproduction of the *Bradyrhizobium japonicum* c-Type cytochrome subunits of the cbb3 oxidase in *Escherichia coli*. Biochem. Bioph. Res. Co. *251*, 744–747. https://doi.org/10.1006/bbrc.1998.9549.

5. Jensen, H.M., TerAvest, M.A., Kokish, M.G., and Ajo-Franklin, C.M. (2016). CymA and exogenous flavins improve extracellular electron transfer and couple it to cell growth in Mtr-expressing *Escherichia coli*. ACS Synth. Biol. *5*, 679–688. https://doi.org/10.1021/acssynbio.5b00279.

6. Dundas, C.M., Walker, D.J.F., and Keitz, B.K. (2020). Tuning extracellular electron transfer by *Shewanella oneidensis* using transcriptional logic gates. ACS Synth. Biol. *9*, 2301–2315. https://doi.org/10.1021/acssynbio.9b00517.
